# Unraveling non-target screening variability for LC-HRMS data: a chemometric comparative analysis of river water samples impacted by treated wastewater

**DOI:** 10.1007/s00216-025-05966-1

**Published:** 2025-06-25

**Authors:** Felix Drees, Maryam Vosough, Torsten C. Schmidt

**Affiliations:** 1 Instrumental Analytical Chemistry, University of Duisburg-Essen, Universitätsstraße 5, Essen, 45141 Germany; 2 Centre for Water and Environmental Research, University of Duisburg-Essen, Universitätsstraße 5, Essen, 45141 Germany; 3 Department of Clean Technologies, Chemistry and Chemical Engineering Research Center of Iran, P.O. Box 14335-186, Tehran, Iran; 4 IWW Water Centre, Moritzstrasse 26, Mülheim an Der Ruhr, 45476 Germany

**Keywords:** Non-target screening, LC-HRMS, Chemometrics, Aquatic ecosystem, ROIMCR, MZmine3

## Abstract

Non-target screening (NTS) using liquid chromatography–high-resolution mass spectrometry (LC-HRMS) is crucial for identifying organic compounds and patterns in complex environmental samples. However, discrepancies in data processing between peak-picking algorithms (“feature profile” approaches) remain a critical challenge in achieving consistent and reproducible results. An alternative approach employs multi-way chemometric methods to directly produce “component profiles,” allowing efficient decomposition and evaluation of LC-HRMS datasets, often after data compression. This study compares two distinct NTS workflows—MZmine3 and regions of interest multivariate curve resolution-alternating least squares (ROIMCR)—using surface water samples from a large-scale mesocosm experiment receiving wastewater effluent over a 10-day exposure period. Samples were analyzed in both positive and negative ionization modes to assess workflow influence on subsequent multivariate analyses. ANOVA simultaneous component analysis (ASCA) quantified treatment and temporal effects, identified dynamic patterns, and prioritized relevant features, while partial least squares discriminant analysis (PLS-DA) discriminated effect-based classes and highlighted significant chemical components and features. Results demonstrated that both workflows significantly differentiated treatment and temporal effects but exhibited different characteristics. Temporal variation dominated through implementing ROIMCR (35.5–70.6% variance), whereas MZmine3 showed comparable contributions from time (20.5–31.8%) and sample type (11.6–22.8%). MZmine3 showed an increased sensitivity to treatment effects but increased susceptibility to false positives. ROIMCR provided superior consistency, reproducibility, and temporal clarity but lower treatment sensitivity. Additionally, workflow agreement diminished with more specialized analytical objectives and prioritized features through implementing multivariate chemometric approaches, highlighting the non-holistic capabilities of individual NTS workflows and the potential benefits of their complementary use.

**Graphical Abstract:**

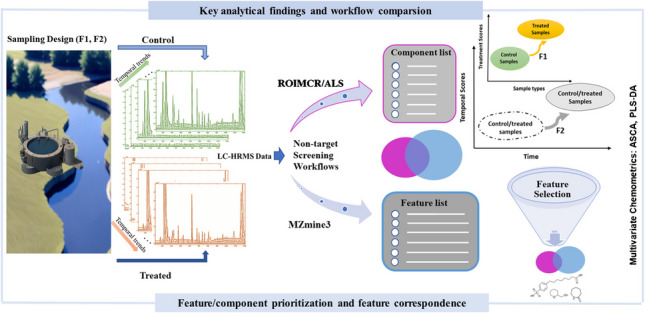

**Supplementary Information:**

The online version contains supplementary material available at 10.1007/s00216-025-05966-1.

## Introduction

Non-target screening (NTS) has emerged as an indispensable analytical approach for identifying and characterizing unknown compounds, especially in environmental studies where the detection of unforeseen pollutants is crucial for understanding complex chemical mixtures. This methodology leverages advanced liquid chromatography coupled with high-resolution mass spectrometry (LC-HRMS) to generate comprehensive datasets. These datasets, characterized by their complexity and volume, necessitate the implementation of sophisticated data processing tools to extract meaningful insights into chemical compositions, patterns and interactions [1, 2]. NTS is particularly valuable for addressing multifactorial questions about study design, sampling influences, and the intricate dynamics of environmental systems [3].

Effective initial data processing is fundamental to NTS, as it converts raw analytical outputs into structured and interpretable datasets. This process involves critical steps such as feature extraction and alignment, noise filtering, feature grouping, and annotations. The primary objective is to produce a comprehensive and accurate “peak list” that reliably captures as many molecular features as possible in the environment while minimizing the inclusion of noise and artifacts. Publicly available “feature profile” (FP) packages such as XCMS [4], MZmine [5], OpenMS [6], and enviMass [7] are frequently utilized for initial raw data analyses, while more comprehensive tools like PatRoon [8] and InSpectra [9] are available for deeper data exploration and interpretation [10]. Among these, MZmine3 [11] has gained significant traction due to its flexibility and integration capabilities, making it a proper choice in environmental NTS workflows. However, it is known that different NTS algorithms, even with similar processing parameters, often generate varying peak lists, with notably low levels of agreement, which can lead to significant differences in chemical or environmental interpretations through downstream data processing steps [12–18].

A powerful alternative to feature profiling (FP) is multi-way processing of LC-HRMS datasets for direct components recovery. In this context, the initial data from each LC-HRMS experiment, comprising scans of unequally spaced masses, must initially be compressed to form a data matrix by utilizing the regions of interest (ROI) search [19] or binning approach [20]. Given that a set of LC-HRMS data for several samples can be viewed as three-dimensional array, multi-way decomposition methods can be effectively employed to directly resolve all signal components carrying systematic variation from complex mixture. Due to its remarkable flexibility, multivariate curve resolution-alternating least squares (MCR/ALS) is a commonly used multi-way decomposition approach to process LC-HRMS data. ROIMCR performs bilinear decomposition of augmented ROI-based matrices, and as a result, three matrices of “component profile” (CP) are generated: (i) resolved “pure” LC profiles, (ii) their mass spectral counterpart, including precursor ions, their associated isotopic peaks, fragments and adduct peaks, and (iii) area under resolved LC profiles as quantification scores. Therefore, data dimensionality can be reduced, decreasing the risk of incomplete componentization and missing compounds that cannot be detected by feature-based peak detection [10, 21]. During recent years, MCR/ALS has been employed for multi-way modeling of LC-HRMS data for some NTS workflows in different contexts of pollution monitoring in aquatic environments, fused modeling of LC-MS^1^ and LC-MS^2^ datasets in water samples [10], environmental metabolomics [22, 23], and wastewater proteomics [24].

Each of these preprocessing workflows, however, has its own pros and cons, and as far as we know, a thorough comparison has not been performed yet between FP packages and CP approaches in a NTS scenario. Such comparisons are crucial for understanding the qualitative and quantitative differences in how these methods extract either a holistic or prioritized subset of chemical spaces.

This study evaluates and compares two NTS data processing approaches—MZmine3 (FP-based) and ROIMCR (CP-based)—within a structured experimental framework, utilizing a mesocosm setup of river water partly receiving treated wastewater (TWW) from a municipal wastewater treatment plant. Here, we assess the impact of wastewater effluent exposure (treatment) and temporal dynamics over a 10-day period. Multivariate statistical methods, including analysis of variance simultaneous component analysis (ASCA) and partial least squares discriminant analysis (PLS-DA), were employed to evaluate treatment and time impacts, and to prioritize features with high discriminatory power. By comparing the capabilities and limitations of FP- and CP-based methods, this work aims to elucidate their influence on chemical feature prioritization and interpretation. These insights are crucial for guiding methodological decisions in NTS studies and enhancing the reliability of environmental monitoring frameworks.

## Methods

### Samples and data collection

**Fig. 1:**
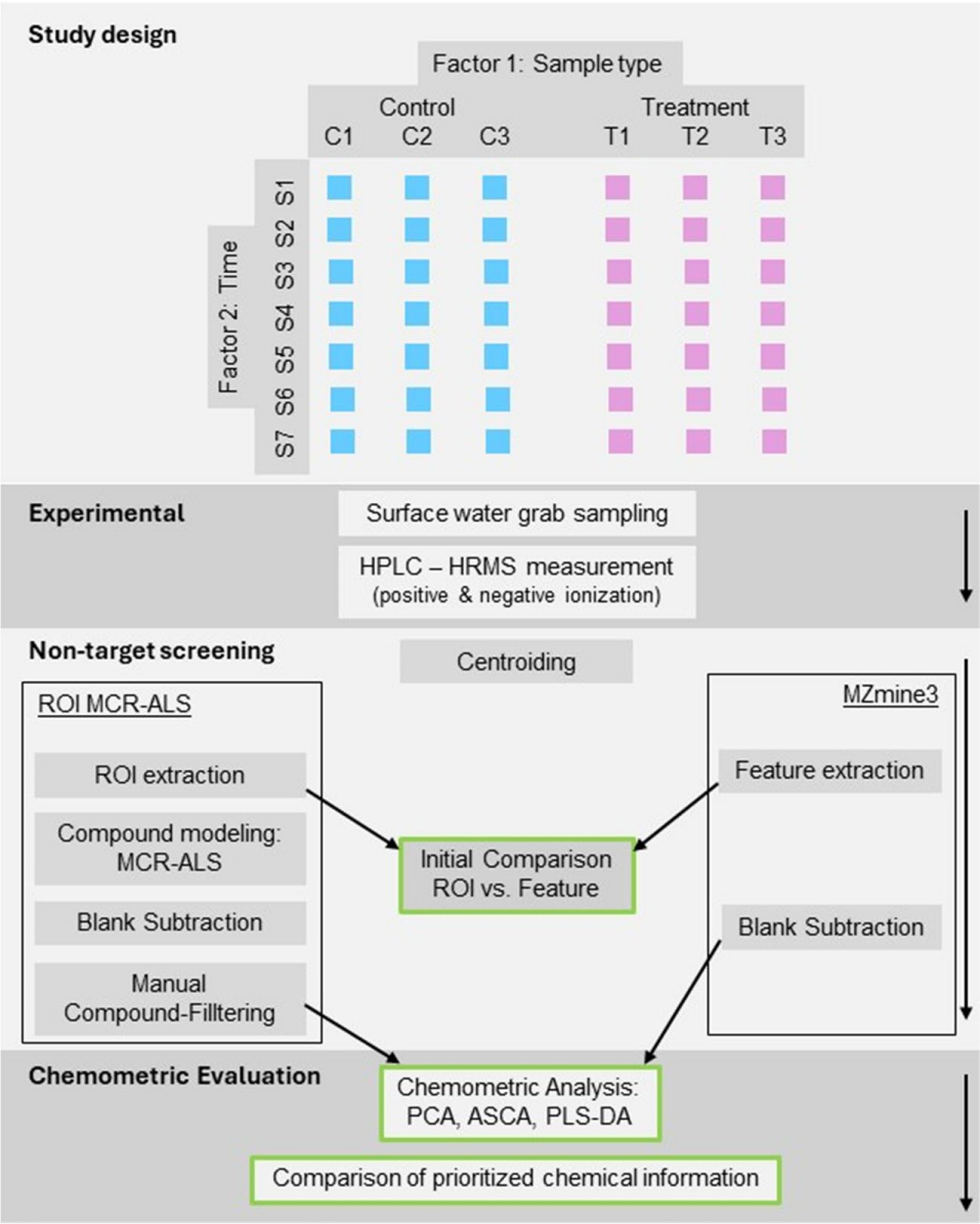
Overview of the presented study, including study design, experimental, non-target screening, and chemometric analysis. Abbreviations: HPLC-HRMS, high-performance liquid chromatography–high-resolution mass spectrometry; PCA, principal component analysis; ASCA, ANOVA simultaneous component analysis; PLS-DA, partial least squares discriminant analysis

Figure 1 provides a graphical overview of our work including information on study design, experimental setup, non-target screening approaches, and subsequent chemometric analysis.

### Study design, sampling, and instrumentation

River water samples from two triplicated large-scale mesocosm setups were systematically analyzed under two experimental conditions: sample type (factor 1) and collection time (factor 2). The dataset included 45 water samples—21 control, 21 treated, and 3 blank—and 8 quality control samples spiked with 11 chemical compounds to optimize processing parameters and ensure measurement stability (see Electronic Supplementary Material Table SI-1). Samples were collected at seven time points over a 10-day period (1 h, 12 h, 1 day, 2 days, 4 days, 7 days, and 10 days) from the experimental setups. The control triplicates (C1, C2, C3) consisted solely of river water, while the treatment triplicates (T1, T2, T3) contained a mixture of two-thirds river water and one-third treated effluent from a municipal wastewater treatment plant. All samples were spiked with internal standards (ISs) at a concentration of 1 µg/L prior to analysis (Table SI-2) [25]. After collection, the samples were filtered using a 0.2-μm polycarbonate filter (Cytiva, Nucleopore, Freiburg), and 1 mL of each filtered sample was transferred into amber vials. From these, 20-µL aliquots were injected into the Orbitrap LC–MS system for analysis. Samples were analyzed using LC-HRMS with a total chromatographic run time of 30 min and a flow rate of 0.3 mL/min. Additional instrumental details, including the gradient elution program, are provided in Figure SI-1 and Tables SI-3 and SI-4.

### Data pre-processing

All raw chromatographic data were acquired in profile mode using Xcalibur software (Thermo Fisher). The data were then centroided using the msConvertGUI (Table SI-5) software [26], with the file format converted to.mzXML [26]. For the ROI approach, the converted chromatograms were imported into MATLAB environment using MSroi GUI app [27] as multiple chromatographic runs and arranged as a column-wise augmented data matrix. MCR/ALS modelings were conducted using MCRALS2.0 toolbox, available at http://www.mcrals.info. MZmine3 (version 3.9.0) processing software was employed by direct import of.mzXML chromatographic data for positive and negative ionization mode (IM) in separate workflows. Identically in both data processing pipelines, first 330 s, which chromatographically correspond to signals associated with the injection peak, were excluded from the analysis. QC samples, containing 11 chemical standards and/or 4 isotopic labeled standards (Tables S-1 and 2), were used to optimize the parameters of the ROI and MZmine3 feature extraction to ensure that all target substances were detected.

#### MZmine3 parameters

We followed the NTS workflow for LC–MS data in MZmine3 [28], including mass detection [29], chromatogram building, deconvolution [30], isotope analysis, and alignment [31]. The Automated Data Analysis Pipeline (ADAP) chromatogram builder was used for extracted ion chromatogram (XIC) construction. Since XICs might be multi-modal, the local minimum resolver module was applied. Thereafter, sample-wise feature lists were aligned using the “Join aligner” module. A mass tolerance of 0.005 Da and maximum retention time ambiguity of 0.3 min were accepted. With the intention of extracting low abundant features, which might fluctuate around our set parameters, we made use of the gap-filling approach. Blank subtraction was performed using a defined fold change threshold to account for system blanks. All details regarding software settings are provided in Tables SI-6 and 7. As a result of this process, a data table is produced with the selected features (identified by their m/z and retention time values) in the rows and the areas of these features in the columns.

#### ROI parameters and MCR/ALS processing

For ROI extraction, the signal intensity threshold and mass tolerance parameters were set as for MZmine3 (SI). In addition, 30 consecutive scans were optimal for detecting all target compounds and maximizing mass overlaps with MZmine3. Through this step, a global data matrix was generated for each IM. In the matrix, the rows represent the product of elution times and samples, while the columns represent the number of ROIs. The global data matrices were then segmented into chromatographic regions and individually subjected to MCR/ALS analysis without further pre-processing. Bilinear factor decomposition of each global data matrix **D**_aug_ containing K matrices was performed as:

1 $$\begin{array}{cc}D_{aug}=\begin{bmatrix}D1\\D2\\D3\\\cdot\\\cdot\\\cdot\\Dk\end{bmatrix}=\begin{bmatrix}C1\\C2\\C3\\\cdot\\\cdot\\\cdot\\Ck\end{bmatrix}\;S_{MS}^T+\begin{bmatrix}E1\\E2\\E3\\\cdot\\\cdot\\\cdot\\Ek\end{bmatrix}=C_{aug}\;S_{MS}^T+E&K=53\end{array}$$

where the rows in matrix **D**_aug_ contain the recorded MS^1^ spectra as a function of time, the columns of **C**_aug_ contain the elution time profiles of the N selected compounds involved in the process for all individual sub-matrices, and the columns of **S**^T^ represent their corresponding pure MS^1^ spectra. Model residuals are expressed as a matrix **E** (Eq. 1). At the end of this step, the resolved chromatographic profiles, their corresponding peak areas (as quantification surrogate scores), and mass spectral peaks (groups of multiple m/z features corresponding to each chromatogram) across different LC windows (as **C**_aug_, **A**_aug_, and **S**_MS_ matrices) were retrieved [23] (see more details in SI-4). Subsequently, **A**_aug_, with blank/water samples and resolved MCR/ALS components was cleaned to remove non-mesocosm-related sample components. In this case, blank correction was done in the same way as in MZmine3. Components related to background signals and irrelevant chromatographic peaks were excluded using the shape criteria and consistency across replicate scans [32].

#### Multivariate data processing using PCA, ASCA, and PLS-DA

Statistical assessments of the final data matrices from MZmine3 and ROIMCR approaches were conducted by analyzing peak areas following initial data pre-processing steps, including logarithmic transformation and total area normalization. As a first explorative step, PCA was applied to understand the inherent structure of all non-target datasets. Subsequently, ASCA was then used to assess the statistical significance of factors (F1: sample type, F2: time) and their interactions (F1 × F2) through matrix decomposition, with permutation tests (1000 times) validating the models. Due to appearing outliers, the experimental design became unbalanced, necessitating ASCA+ for unbiased type III sum-of-squares calculations [33]. The selection of significant features was carried out by assessing the robustness of the loading values, using bootstrapping test (*p* < 0.05). Only the variables with a *p*-value lower than 0.05 were considered statistically significant in ASCA models. Finally, PLS-DA was implemented on ROIMCR and MZmine3 data matrices in both ionization modes. We developed two classification models for each data matrix, guided by our previous findings [25] and results from ASCA analyses. The labeling of samples in supervised models was based on the significant impact of the treatment effect (control vs. treatment samples) and the discriminative patterns observed in temporal scores in ASCA models. Consequently, we compared binary classification models along the time dimensions in S1 to S4 and S5 to S7 time frames. PLS-DA models were developed using five-fold Venetian blind cross-validation, and their statistical significance was confirmed through 200 random permutations to prevent overfitting. Based on the generated models, variable importance on projection (VIP) scores were used to identify the most relevant variables [34]. More details regarding implemented methods are provided in SI-5.

PCA, PLS-DA, and ASCA modeling were performed by using PLS Toolbox 8.9.1 (Eigenvector Research Inc., Wenatchee, WA, USA) working under MATLAB (The MathWorks, Natick, MA, USA). The MATLAB MEDA toolbox, available on GitHub, was used for bootstrapping in ASCA models. Figures were created with OriginLab (Pro) 2022b, and Venn diagrams were generated using the “VennDiagram” app (version 1.10) from OriginLab’s File Exchange. Public libraries such as mzCloud (https://www.mzcloud.org) and PubChem (https://pubchem.ncbi.nlm.nih.gov) were searched to identify prioritized MCR compounds. Using Schymanski’s scheme [35], tentatively identified features have been classified.

#### Comparison of NTS results

In an initial step, we compared extracted ROIs and MZmine3 features using a 0.005-Da mass tolerance, focusing on mass correspondence due to challenges in assigning reliable retention time (RT) values. The ROI method includes artifacts and baseline features with poorly defined RTs, while falsely detected features in MZmine3 often suffer from inconsistent RT assignment (especially for electric spikes, noisy or distorted peaks). In emphasizing mass accuracy, a practical starting point for optimizing parameters through correspondence has been established, while acknowledging the limitations of excluding RT from matching criteria. The ROIMCR approach necessitates careful evaluation of all detected features and their prioritization in alignment with FP methods. This requirement stems from its unique componentization workflow, where ROI features are grouped into components through bilinear factorization. Following MCR modeling and filtering non-relevant MCR components, the resolved mass spectral profile for each MCR component was unfolded into its m/z features and assigned its RT. To establish the correspondence between the feature list provided by MZmine3 and the ROI-derived mass features associated with MCR components, we employed retention time and mass alignment criteria (0.3 min and 0.005 Da). Similarly, to assess overlaps between prioritized features and enable a meaningful comparison with the MZmine3 approach, we selected ROIs that were characteristic of a prioritized component based on its resolved mass spectrum via the MCR/ALS approach. Specifically, ROIs contributing more than 5% of the m/z base peak intensity in each mass spectrum were included in the comparison.

## Results and discussion

**Fig. 2:**
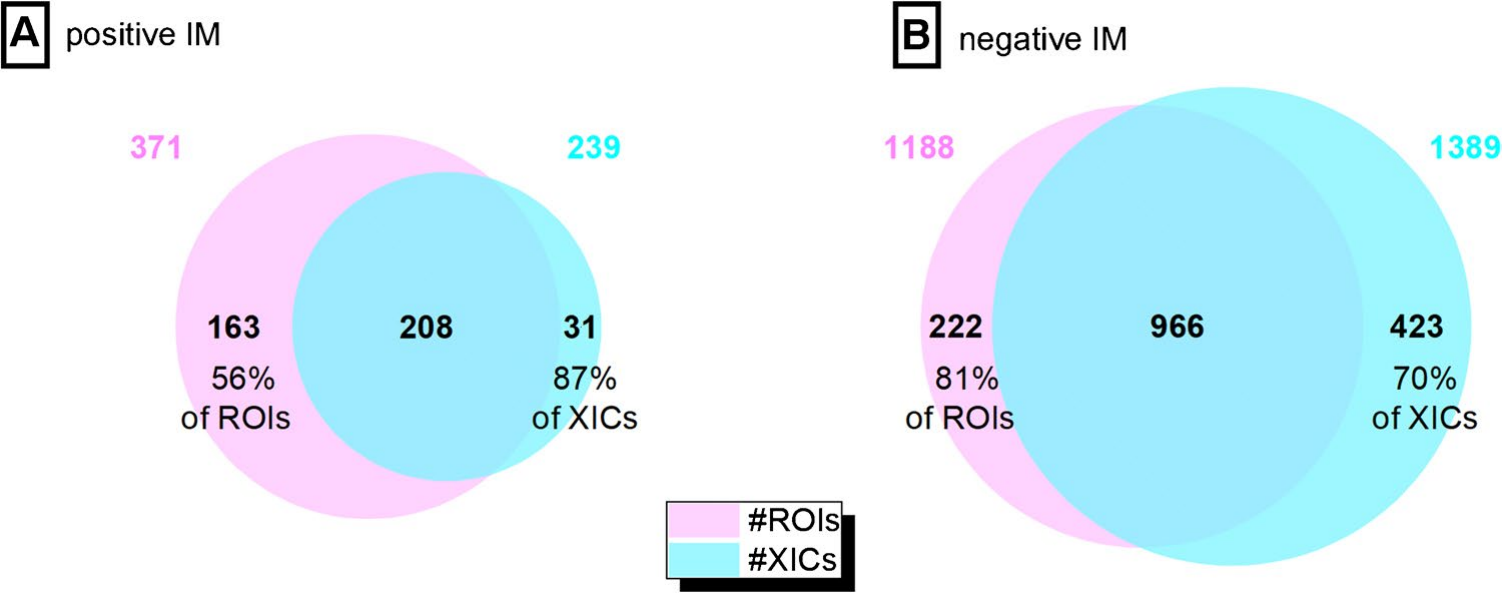
Visualizing initial correspondence analysis between extracted ion chromatograms (XICs) by MZmine3 and regions of interest (ROIs) in both positive (**A**) and negative (**B**) ionization modes (IMs) for QC samples

This work compared the ROI and ROI-based MCR/ALS method with the commonly used peak-picking algorithm MZmine3. An initial parameter setting was necessary to use equivalent values for each approach. Up to the step of XIC building in MZmine3 using ADAP, both NTS strategies apply nearly equivalent filters, including signal intensity thresholds, mass error tolerances, and the number of consecutive retention times. With regard to the later parameter and due to fact that ADAP algorithm uses some rigid criteria that can overlook real features with suboptimal peak shapes, we conducted a comparative analysis using QC samples to infer the impact of this parameter. Here, we used target compound recovery and the total number of shared features serving as the benchmark for selecting optimal occurrence frequency setting. As can be appreciated from Tables SI-8 and 9, while recovery of target compounds in each method remains as 100%, the shared feature rating is enhanced while minimum occurrence frequencies are set to 30 and 5 for ROI and MZmine3, respectively. Any variation from these values for each approach would lead to missing some targets or deflating shared feature space between two methods or increasing distinct ROI features. The optimal point visualized in Fig. 2 reveals that the noticeable part of extracted ROIs and XICs are mutually consistent. In positive IM, 56% of ROIs align with XICs, and 87% of XICs align with ROIs. In negative IM, 81% of ROIs match XICs, and 70% of XICs match ROIs. The difference observed between MS ionization modes highlights that, in addition to initial parameter settings, sample composition plays a crucial role in influencing the variation between non-targeted methods in capturing both chemical and non-chemical features [36–39].

For the full dataset of 53 samples, a comparison was made between 3011 ROIs and 2584 features in the positive IM, and 5941 ROIs and 8959 features in the negative IM. In these comparisons, 40% and 57% of the ROIs were covered by features for positive and negative IMs, respectively, while 88% and 86% of the features were covered by ROIs, which is in agreement with a broad perspective of ROI compared to the MZmine3 approach, which incorporates numerous additional filters and hard criteria.

### Resolving process for LC-HRMS data using MCR/ALS

The integration of ROI data compression and curve resolution strategy based on MCR/ALS effectively addressed several critical challenges in NTS studies [10, 21, 40]. These challenges included highly overlapped chromatographic profiles, retention time shifts, background drift, signal artifact resolution, and substantial data size reduction. Subsequently, twelve sets of column-wise augmented LC-HRMS data matrices encompassing river water samples, QC samples, and blank samples were simultaneously decomposed using extended MCR/ALS models. The MCR/ALS modeling facilitated the identification and extraction of all chemical factors causing systematic variations across the datasets. Figure SI-2 displays resolved chromatograms and MS^1^ spectra for the second region of LC-HRMS data (positive IM), including chemicals, and irrelevant peaks [21] in water samples. This figure also illustrates the recovery of the carbamazepine-D8 (IS) and the treatment-relevant component cp186. The models achieved a minimum explained variance of 97% and a maximum lack of fit (LOF) value of 7.3%. Consequently, a total of 203 and 147 MCR/ALS components in positive and negative IMs, respectively, were used to elucidate variance across the dataset, capturing all detected species, solvent contributions, noise, and background signals. To refine data quality, matrix cleaning process (for **C**_aug_, **A**_aug_, and **S**_MS_ matrices) was implemented and validated empirically to eliminate noisy signals, background components/artifact signals, and MCR components lacking reliable chromatographic shapes [21, 32, 40]. Lastly, 101 and 75 modeled components were retained for positive and negative IM, respectively. In other words, the bilinear decomposition of the data matrices showed that nearly 50% of the resolved components—each associated with a varying number of ROIs—were identified as false positives. The peak area matrices with dimensions 42 × 101 (positive IM) and 42 × 75 (negative IM) were subsequently used for multivariate analysis.

### MZmine3 data reduction

The further data compression in the MZmine workflow was carried out in a similar manner as described for MCR/ALS. Starting with 2584 features extracted in positive IM and 8959 in negative IM, the number of features was reduced to 1929 and 4714 respectively after excluding features, which chromatographically were associated with the injection peak. Following the previously described blank subtraction, these numbers further decreased for the 42 core samples that were ultimately considered for chemometric analysis, resulting in 530 features in positive IM and 3460 features in negative IM. Consequently, the final data matrices are in the dimensions of 42 × 530 and 42 × 3460. These features are not all independent compounds, since some could be adducts or isotopic masses derived from a single compound, while others could be false positives such as non-filtered artifact signals. In this case, no further attempt was made to componentize features or further filter out features using peak attribute criteria. An overview on the matrix dimensions throughout the current work is shown in Figure SI-3.

### Analysis of further reduced NTS datasets: overlap and divergence

Following the data reduction process, features decreased by 25% in positive IM and 47% in negative IM. ROIs experienced smaller reductions, dropping by 15% (3011 to 2542) in positive IM and 20% (5941 to 4742) in negative IM. Despite this, 84% of features in positive IM and 87% in negative IM remain covered by ROIs, while ROI coverage by features declined from 40 to 36% and 57 to 31%, respectively. Consequently, 64% and 69% of ROIs not detected by MZmine3 in positive and negative IM, respectively, may be considered false negatives. These are likely due to restrictive shape-based filtering criteria (e.g., peak sharpness), complex overlapping signals, or non-ideal slopes [41]. As a soft-modeling approach, MCR/ALS effectively addresses the issues like non-ideal chromatographic shapes for true chemicals, shape difference across different samples, as well as chromatographic shifts. Generally, in extreme coelution cases, especially when a true component embedded under a irrelevant signal, probable resolution uncertainty would lead to appearance of false (m/z ROI) positives [42]. However, we found that these uncertainties were considerably limited for the selected MCR components, primarily due to substantial variability in component concentrations (pronounced compositional changes), which effectively reduced concentration collinearities [40], high resolution of MS features, and the multi-set structure of data decomposition [43, 44]. Typically, false negatives in ROIMCR may result either from undetected ROIs—due to the implemented ROI settings—or from a suboptimal resolution process. The latter can occur when low-abundance pollutants are obscured by dominant matrix compounds across all sample types. These components contribute minimally to overall variance and may therefore be overlooked during the optimization process. Alternatively, extremely trace compounds may remain in the MCR/ALS residuals due to an underestimation of the number of components. However, proper validation and careful optimization of the ROIMCR workflow, particularly when applied to narrow chromatographic windows, can substantially reduce the risk of false negatives [40, 45].

### Multivariate modeling of environmental water samples

#### Initial data exploration

PCA revealed that the first three principal components cover a varying amount of variance (51.3–69.3%) for the NTS datasets. For MCR/ALS, negative IM, the first three PCs explain 51.3% of the total variance. This is relatively lower compared to the MCR/ALS, positive IM, where it is 69.3%. In MZmine3, both ionization modes captured a comparable amount of total variance, 54.0% and 51.2%, respectively. In the PC1-PC2 subspace, control and treatment samples were clearly distinguished for MZmine3 and MCR/ALS (negative IM), but not for MCR/ALS results with a positive IM, as a result of higher captured temporal variability (Figure SI-4). Moreover, both approaches display a different degree of time trend variability. A more elaborate inference of pattern of the temporal dynamic has been presented in Figure SI-5.

#### Assessment of factors and features by ASCA

The study design was incorporated into the ASCA modeling of all datasets to better highlight the previous observations, sample type, and time effects. Because of the removal of outlying samples, the resulting experimental design is unbalanced and ASCA+ analysis allows us to determine which experimental factor significantly influences the underlying data structure. Respective results are shown in Table 1.

**Table 1:** ASCA results for MCR/ALS and MZmine3 datasets in positive and negative IM. Significance and partitioning of the total variance into individual terms corresponding to factors and potential interaction

Dataset	Factor	Percentage of variation^a^	Significance (*p*-value)
MCR/ALS, negative IM	Sample type^b^	13.3	< 0.001
	Time^c^	35.5	< 0.001
	Sample type × time	8.3	0.94
	Residuals	42.9	
MCR/ALS, positive IM	Sample type	2.4	< 0.001
	Time	70.6	< 0.001
	Sample type × time	4.6	0.87
	Residuals	22.4	
MZmine3, negative IM	Sample type	22.8	< 0.001
	Time	20.5	< 0.001
	Sample type × time	10.9	0.27
	Residuals	45.9	
MZmine3, positive IM	Sample type	11.6	< 0.001
	Time	31.8	< 0.001
	Sample type × time	8.0	0.95
	Residuals	48.6	

^a^Percentage of variation expressed as the sum of squared deviations from the overall mean

^b^Sample type factor with two levels: controland treatment

^c^Time factor with seven levels: 1, 12, 24, 48 (h), 4, 7, and 10 (d)

**Fig. 3:**
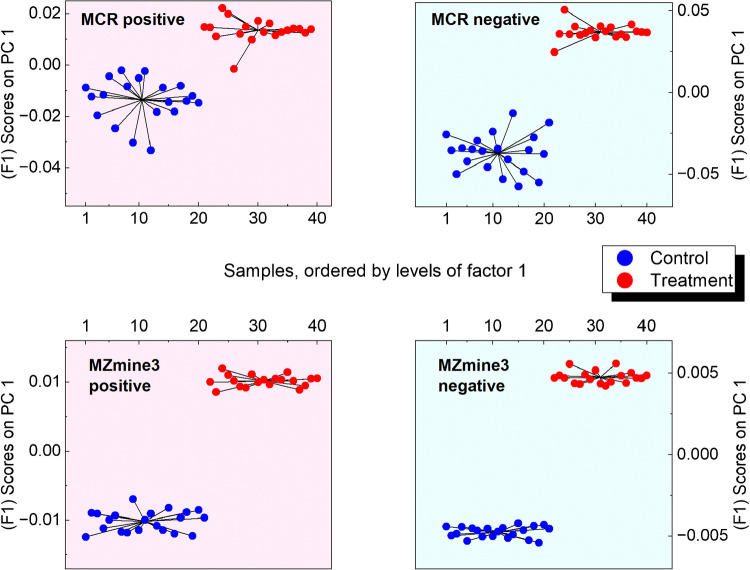
Scores on PC 1 (factor 1) obtained from ASCA of feature and component list by MZmine3 and MCR/ALS in both IMs. The color code indicates sample types, control (blue) and treatment (red)

The permutation test shows that the experiment design-based factors, sample type and time, are assessed as significant in each dataset (*p*-value < 0.001). Conversely, their interaction is consistently deemed as not significant (*p*-values > 0.05), indicating that the interactive effect of sample type and time on the variance is not as pronounced or consistent as the individual effects. Residual variance, representing unexplained variation not captured by the model, is higher in MZmine3. This implies a greater influence of noise or other non-systematic variability in the MZmine3 datasets. Regarding the percentage of variation explained by each factor, time generally emerges as the dominant factor across both NTS approaches. This effect is particularly evident in positive IM, where time accounts for over 70% of the variance in ROIMCR data and approximately 32% in MZmine3. This suggests that time-dependent changes are more pronounced or better captured in positive IM. In both workflows, sample type distinction explains a relatively smaller portion of the variance. However, the magnitude of this effect is much greater in negative IM. For MZmine3, the impact of sample type is more substantial in negative IM (22.8%) compared to positive IM (11.6%). However, ROIMCR positive IM revealed capturing a subtle treatment impact which further support previous findings by PCA. Figure 3 visualizes the treatment effect (F1) using ASCA score plots for all data matrices.

**Fig. 4:**
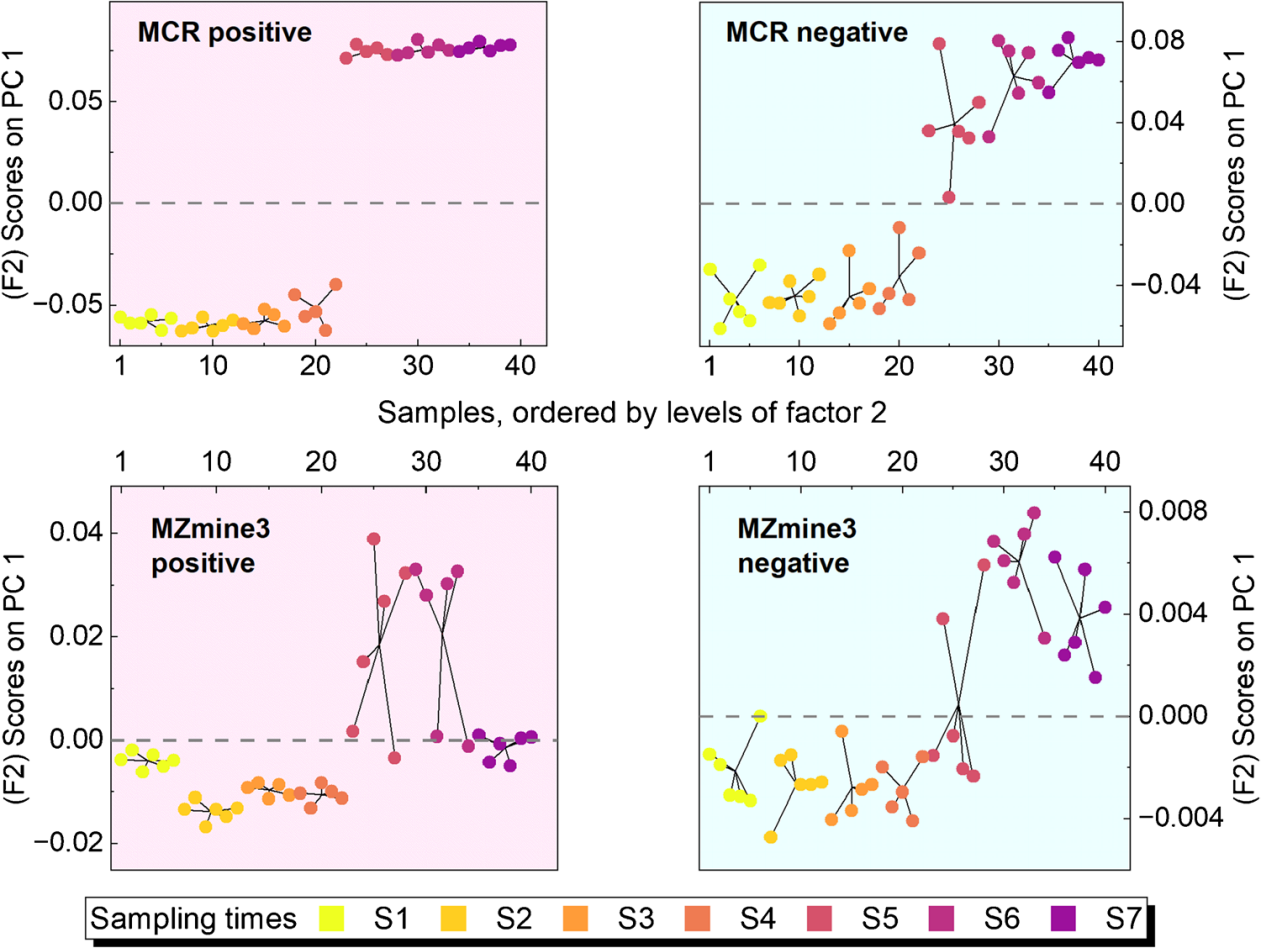
Scores on PC 1 (factor 2) obtained from ASCA of feature and component list by MZmine3 and MCR/ALS in both IMs. The color code represents the sampling time series, while the background color denotes positive (blush) or negative (light-blue) IM. Sampling times are S1:1 h, S2:12 h, S3:1 day, S4:2 days, S5:4 days, S6:7 days, and S7:10 days

Figure 4 shows F2 scores on PC1 for samples over sampling times S1 to S7, clearly indicating distinct temporal patterns and clustering of samples at different times, suggesting significant changes in the water sample composition or concentration of analytes over time. ROIMCR displays more consistent temporal groupings, suggesting a higher capability to capture dynamic patterns. In particular, two subclasses can be distinguished between S4 and S5 based on PC1 scores. ROIMCR results present this transition more clearly. In contrast, MZmine3 scores show weaker separation over time, with greater overlap across sampling points and reduced clustering, indicating limited reproducibility at certain points. This might imply that MZmine3 data is less effective at capturing or distinguishing temporal trends in the current experimental setup. However, although statistical significance was confirmed for the MZmine3 dataset—as it was for the ROIMCR dataset—the distorted score trends suggest a possible influence of false positive features retained in the MZmine3 feature list and propagated through downstream analysis. Additionally, inconsistencies in the reproducible recovery of features in some replicate samples (e.g., at time point S5, which exhibited sharp chemical changes) or at time points with more complex matrix composition may have contributed to this variability [46]. On the other hand, a high overlap between the first and last temporal point (with dense intra-group variation) in MZmine3 positive IM data might indicate over-filtering of key features that distinguish extreme temporal points, potentially leading to false negatives. Furthermore, the interaction scores revealed no discernible pattern related to the interaction between factors; consequently, the corresponding plot was not included.

The loading values of ROIMCR components and MZmine3 features were analyzed for their contribution to the experimental factors. After bootstrapping the ASCA loadings (Figure SI-6), those components and features with statistically significant contributions (*p* < 0.05) to the observed variability were identified. The results showed that different subsets of components and features were associated with each factor effect. Additionally, the degree of concordance between prioritized ROIs and features varied depending on the experimental factor and ionization mode. Specifically, only 15% and 8% of MZmine3 features in positive IM corresponded to the prioritized ROIs, for F1 and F2, respectively. In contrast, this correspondence increased to 72% and 50% in negative IM. From the ROIMCR perspective, only 10% and 2% of prioritized ROIs matched prioritized MZmine3 features in positive IM, whereas this increased to 44% and 51% in negative IM. The absolute number of prioritized features and ROIs varied with the ionization mode, as detailed in Table SI-10. Figure SI-7 gives the ASCA loading plots for PC1 of temporal effect (F2) obtained for ROIMCR components and MZmine3 features. Here, we exemplarily show some common and unique features related to each of NTS workflow. Since the componentization step is not performed in MZmine3, a single prioritized ROIMCR component may correspond to multiple m/z features, including isotopic peaks or adducts of the same compound. As shown in this figure, common features between the two workflows show well chromatographic peak shapes with moderate to strong peak intensities (e.g., ROIMCR cp1 and cp6). In contrast, false negative features in MZmine3 are characterized by low-intensity XICs (e.g., ROIMCR cp84) or peaks with a trailing shape (e.g., ROIMCR cp66) [41]. Additionally as an example of a false negative peak for ROIMCR, m/z index 325 ([M + H]^+^ = 472.4370) is shown. This feature was not detected during ROI step due to an insufficient number of consecutive appearances.

#### PLS-DA models and feature selection

Discrimination of water samples in each of ROIMCR and MZmine3 peak area data matrices, according to the effects derived by ASCA was then investigated. To this end, several PLS-DA models were constructed using tentatively categorized sample groups. For each IM, two binary classification models were developed: one distinguishing between control and treated water samples, and another distinguishing between early (S1–S4) and later (S5–S7) sampling time points, regardless of sample type. Additionally, three-class PLS-DA models were constructed for each IM, differentiating among (i) control samples from S1–S4, (ii) treated samples from S1–S4, and (iii) all samples from S5–S7.

Table SI-11 summarizes the model performance parameters of the PLS-DA models. For both MZmine3 and ROIMCR workflows in each ionization mode, samples were distinguished according to the wastewater treatment effect (sample type classification), but also based on temporal differences (time classification). Overall, the two-class PLS-DA models showed lower classification error rates and higher *R*^2^ values than the three-class models, indicating more stable and reliable performance. Specifically, ROIMCR negative IM and MZmine3 models achieved the highest *R*^2^ values. In contrast, the three-class PLS-DA models, particularly for MZmine3 positive IM, showed the highest classification errors and lower *R*^2^ values, suggesting a reduced ability to discriminate between the three categories. ROIMCR in positive IM demonstrated the strongest three-class classification performance, showing high explained variance and improved *R*^2^ values. However, the variability observed across different three-class models suggests this classification strategy is less reliable for further analysis. Given the objective of identifying important discriminatory features and comparing VIPs across MZmine3 and ROIMCR workflows, the two-class PLS-DA models were the preferred choice due to their stronger classification performance, higher reliability, and better consistency across both workflows. A further evaluation of the misclassification cases in the ROIMCR positive IM revealed a significant reduction in sensitivity when distinguishing between control and treated samples at the final temporal points (S6 and S7). This aligns well with previous findings regarding a general subtle treatment effect and a diminished distinction between the two groups as the experiment progressed. Meanwhile, samples relevant to time slots S1-S4 in both IM workflows were clearly distinguished from samples related to time slots S5-S7 with a similar class error of 0.028. This indicates consistency in the temporal classification models, despite differences in the underlying chemical spaces captured by ROIMCR and MZmine3.

**Fig. 5:**
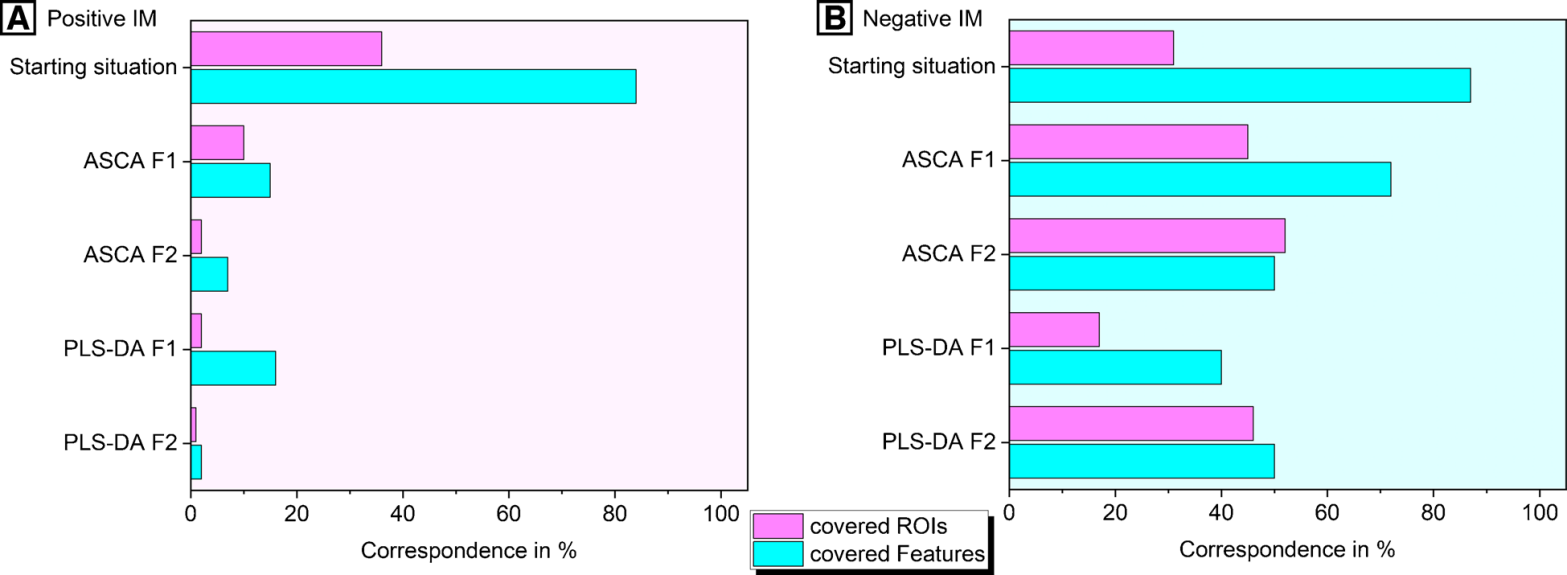
Relative comparison of ROIs and MZmine3 features depending on the analysis step and ionization mode (positive panel **A**, negative panel **B**). The starting situation describes the initially developed ROI and feature matrix. While chemometric analysis in the ASCA+ and PLS-DA approaches focused on components within the ROIMCR framework, only those ROIs contributing quantitatively to 5% or more of a component’s mass spectrum were considered. The correspondence calculation between prioritized ROIs and features included multiple matches, reflecting that a single ROI may contribute to multiple MCR components

For classification purposes, features and ROIMCR components, and their corresponding ROIs, have been prioritized based on their VIP values (> 1). The corresponding comparisons for each factor and ionization mode are shown in Fig. 5. This figure also includes the results of the initial comparison and the ASCA prioritization, offering a comprehensive overview of the correspondence between both methods throughout the study. However, it should be mentioned here, that while we compared the correspondence of feature lists with each other in the current study, the multivariate modeling and prioritization step for ROIMCR is based on recovered components not m/z features. The components are more robust predictors compared to unfolded m/z features due to their increased redundancy and influence in differential analysis approaches.

Based on Fig. 5, it can be concluded that in positive IM, and consistent with the ASCA analysis, the correspondence of features in the PLS-DA results significantly decreased compared to the initial coverage of MZmine3 features by ROIs. Notably, F1-based classifications exhibited an 18% correspondence, while all other classifications retained less than 5%, indicating a substantial loss of selected features. In contrast, the negative IM demonstrated a notably higher correspondence of relevant features in the PLS-DA models (40–50%), in alignment with the ASCA. The observed variability between correspondence ratios of the multivariate methods (regardless of NTS workflow) supports the complementary roles that ASCA and PLS-DA play in identifying significant variables, as reported previously [47]. A comparable trend was observed in ROIMCR, with the negative IM yielding a considerably higher overlap between prioritized ROIs and MZmine3 features. This reinforces the strong dependence of feature prioritization overlap across different NTS workflows on the initial chemical space captured. In the current experimental setup, the number of detected true chemically relevant features in negative mode is substantially higher than in positive mode. As a result, these features are more likely to pass MZmine3’s stringent selection criteria, such as ideal slope (i.e., the smoothness of a peak), and peak shape, leading to improved alignment with ROIMCR components. A generally higher feature quality in negative IM was confirmed, with lower asymmetry (1.66) than positive IM (1.88), indicating better peak shapes. Additionally, lower gap-filling values (1.75 in negative vs. 2.34 in positive) reflect the greater stability and completeness of features detected in negative mode.

#### Comparative evaluation of NTS workflows

Implementing both ROIMCR and MZmine3 on the same LC-HRMS datasets within the current mesocosm setup enabled their evaluation and provided a solid basis for methodological comparison. Although the tuning parameters were adjusted to maximize overlap between the initial feature spaces and ensure a fair comparison, subsequent filtering and downstream processing revealed varying degrees of divergence. This divergence arises from the fact that each workflow captures a subset of true chemical features, governed by its specific settings, and can be characterized by the following key aspects: (i) false negatives in MZmine3: MZmine3 applies strict criteria that may exclude true features with poor peak shapes (e.g., noisy or irregular slopes), potentially misclassifying low-intensity or non-ideal peaks as noise [41]. Furthermore, limited resolution settings may cause closely eluting compounds to be merged into single feature, contributing further to missed detections in complex or noisy datasets; (ii) false negatives in ROIMCR: While ROIMCR is more effective at handling challenging conditions such as chromatographic shifts, coeluted signals, and non-ideal peak shapes, some true features may still be missed during the ROI detection step due to initial threshold restrictions (e.g., scan continuity requirement); (iii) false positives: MZmine3 is also prone to retaining non-relevant or spurious signals, leading to an increase in false positive cases, especially in complex matrices. Among the possible contributors, the ADAP peak detection algorithm and the chromatogram deconvolution strategy are typically the most influential sources of false positives, particularly due to the use of the peak top-to-edge ratio, which may cause non-relevant peaks with relatively high ratios to be falsely identified as true signals [41]. This phenomenon can compromise the quality of downstream statistical analyses and further feature prioritization. In contrast, the MCR/ALS modeling approach used in ROIMCR involves a thorough component validation process, in which non-reliable components—such as broad, bimodal, square-shaped, or highly noisy peaks—are systematically resolved and empirically excluded; (iv) matrix effect and reproducibility: MZmine3 outputs feature intensity values, whereas ROIMCR provides integrated peak areas from “pure” resolved elution profiles. These resolved profiles reduce the influence of noise and background matrix variability, resulting in more stable and reproducible component profiles compared to MZmine3 and allowing for finer temporal resolution of sample trends (Fig. 4). Our findings indicate that ROIMCR generates more reproducible component lists, particularly in datasets with substantial peak overlap and variable matrix interferences. MZmine3, by contrast, exhibited greater variability in feature reproducibility due to its stringent selection criteria, often leading to missed detections in some replicate samples, particularly at challenging temporal points. The exclusion of borderline features can lead to increased false negatives and impair ASCA performance. These methodological differences result in distinct feature/component tables, with varying degrees of overlap between the workflows across positive and negative ionization modes. In the context of the complex and dynamic datasets used in this study, such differences help explain the limited alignment observed between prioritized features across different multivariate models—despite both workflows producing generally coherent statistical outcomes and identifying similar significant factors.

### A brief insight into prioritized components

**Fig. 6:**
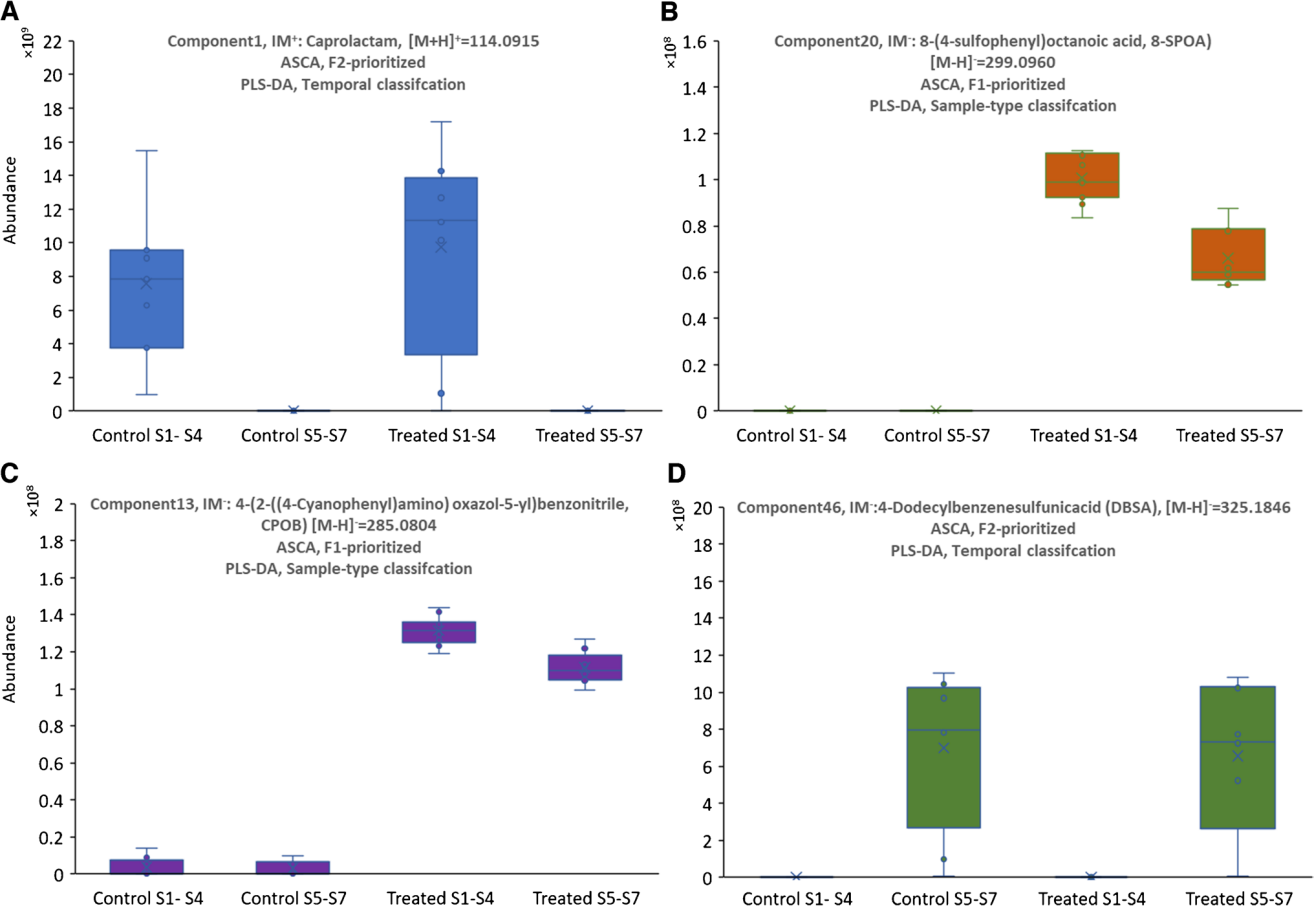
Representation of peak area variations for tentatively identified prioritized components in both NTS workflows. cp1, IM + (CAP) **A**, cp20, IM-, 8-SPOA **B**, cp13, IM-, CPOB **C**, and cp46, IM-, DBSA **D** using ASCA (*p* < 0.05) and PLS-DA VIP > 1

In the following, we will briefly discuss exemplary tentatively identified components (prioritized using both NTS strategies in both chemometrics data processing methods). From the trends visualized in Fig. 6, it is evident that cp1, which is detected in positive IM, [M + H]^+^ = 114.0915 as caprolactam (CAP) is well-suited for distinguishing between the first and second time periods of sampling. Furthermore, it is notable that caprolactam appears to be generally more abundant in the treatment samples, but is also detectable in considerable quantities in the river water. The investigated wastewater treatment plant effluent may represent one of several entry pathways. Caprolactam is produced worldwide on a multimillion-ton scale primarily for the production of nylon. As described [48–50], it can indeed become environmentally relevant and is metabolized by various bacteria. This would likely also explain why caprolactam is no longer detected in samples from the later experimental phase. We also identified the top-ranked NTS cp13 ([M-H]^−^ = 285.0804) and cp20 ([M-H]^−^ = 299.0960) in negative IM, which are associated with wastewater treated samples (F1) as 4-(2-((4-Cyanophenyl)amino) oxazol-5-yl)benzonitrile (CPOB) and 8-(4-sulfophenyl)octanoic acid (8-SPOA). Appearance of CPOB and 8-SPOA as industrial chemical and surfactant compounds in water bodies is often linked to industrial discharge and pharmaceutical residues and may pose risks to aquatic life and microbial communities [51–53]. Also, cp46 (4-dodecylbenzenesulfonic acid, 4-DBSA), with an accurate mass of 325.1846 identified in negative IM, was classified as temporal indicator, evolving as a surfactant byproduct in time slots S5-S7 (4 to 10 days). 4-DBSA can be considered as an intermediate product of 4-sulfophenyldodecanoic acid (detected as cp63, m/z 355.1589) through biodegradation process using microorganisms, including bacteria and algae [54]. A more comprehensive identification of all prioritized and classified pollutants was not within the scope of the present study.

## Conclusion

This study demonstrates the significant impact of wastewater treatment effluent on the chemical diversity and composition of receiving waters. By analyzing surface water samples within a controlled mesocosm setup, we applied two NTS workflows—MZmine3 and ROIMCR—and assessed their effectiveness in detecting temporal and treatment-related variations. Our findings reveal that both methods effectively captured temporal and treatment-related variations, distinguishing class-wise effects. In spite of this general coherence, they differ in their temporal resolution and treatment sensitivity. ROIMCR demonstrated a superior consistency in tracking dynamic changes over time, but a better sensitivity to wastewater impact was realized using MZmine3. In addition, this agreement was achieved despite only a low to moderate overlap in the significant features identified by each NTS approach. While the overlap between prioritized features was limited, the consistency of broader statistical trends across methods highlights their complementary strengths in environmental monitoring. The results reaffirm the importance of minimizing false positives and ensuring the reproducibility of detected features prior to statistical modeling, an especially challenging task in complex environmental matrices and dynamic experimental setups. The findings also emphasize that workflow performance depends heavily on data characteristics—such as ionization mode, MS signal intensity, background signal contributions, temporal variability in chemical composition, and overall data complexity. However, we emphasize that the total number of correspondences between NTS feature lists is not a decisive factor; rather, the relevance, reproducibility, and chemical interpretability of extracted features are critical for robust downstream analysis. Ultimately, this study contributes to a more in-depth understanding of how data processing choices affect the outcomes of NTS workflows and supports the use of complementary approaches like MZmine3 and ROIMCR for robust environmental pollutant detection.

## Supplementary Information

Below is the link to the electronic supplementary material.

ESM 1 (DOCX 995 KB)

## Data Availability

Data are available from the authors upon reasonable request.
