# Supplementary material for: Unraveling non-target screening variability for LC-HRMS data: a chemometric comparative analysis of river water samples impacted by treated wastewater
Source: Anal Bioanal Chem. 2025 Jun 25;417(27):6075–88. doi: 10.1007/s00216-025-05966-1 (PMC12583377; doi:10.1007/s00216-025-05966-1)
Supplement: Supplementary file 1 — (DOCX 995 KB) [file 216_2025_5966_MOESM1_ESM.docx]

Electronic Supplementary Material

**Unraveling Non-Target Screening Variability for LC-HRMS Data:**

**A Chemometric Comparative Analysis of River Water Samples Impacted by Treated Wastewater**

Felix Drees^1,2^, Maryam Vosough*^1,2,3^ and Torsten C. Schmidt^1,2,4^

*^1^Instrumental Analytical Chemistry, University of Duisburg-Essen, Universitätsstraße 5, 45141 Essen, Germany*

*^2^Centre for Water and Environmental Research, University of Duisburg-Essen, Universitätsstraße 5, 45141 Essen, Germany*

*^3^Department of Clean Technologies, Chemistry and Chemical Engineering Research Center of Iran, Tehran, Iran, P.O. Box 14335-186 Tehran, Iran*

*^4^IWW Water Centre, Moritzstrasse 26, 45476 Mülheim an der Ruhr, Germany*

*^*^Corresponding author. E-mail address:* [*maryam.vosough@uni-due.de*](mailto:maryam.vosough@uni-due.de) *(M. Vosough)*

**Table of contents**

**SI-1:** Chromatography and instrumentation

**SI-2:** MZmine3 parameters

**SI-3:** Region of interest (ROI) approach

**SI-4:** MCR-ALS Resolution of ROI LC-HRMS Data

**SI-5:** Multivariate Data Processing

**SI-6:** Initial parameter setting and correspondence

**SI-7:** MCR/ALS resolution: Example

**SI-8:** Overview on data reduction and organization throughout this study

**SI-9:** Supporting results for PCA, ASCA and PLS-DA

## **Chromatography and instrumentation**

A Dionex UltiMate 3000 HPLC system (Thermo Scientific, Bremen, Germany) was used to perform chromatographic separation. The separation involved the use of two mobile phases: Eluent A, which was ultrapure water, and eluent B, which was methanol, both with 0.1% (v/v) formic acid (MS grade). The injection volume was 20 μL. High resolution mass spectrometric (HRMS) detection was carried out using an orbitrap mass spectrometer (QExactive Thermo Scientific, Bremen, Germany) with electrospray ionization in positive and negative mode. The acquisition of full scan HRMS spectra (m/z 80-1000) was performed with a resolution of 70,000, followed by data-dependent MS^2^ scans of the five most intense ions at a resolution of 17,500. Mass calibration was performed using respective calibration solutions (Pierce LTQ Velos Positive/Negative Ions Calibration Solution, Thermo Scientific, Bremen, Germany). Software‑wise, the HPLC was operated using Chromeleon 7.2 (Thermo Scientific, Germany), while the HRMS was run with Xcalibur^TM^ 4.3 (Thermo Scientific, Germany).


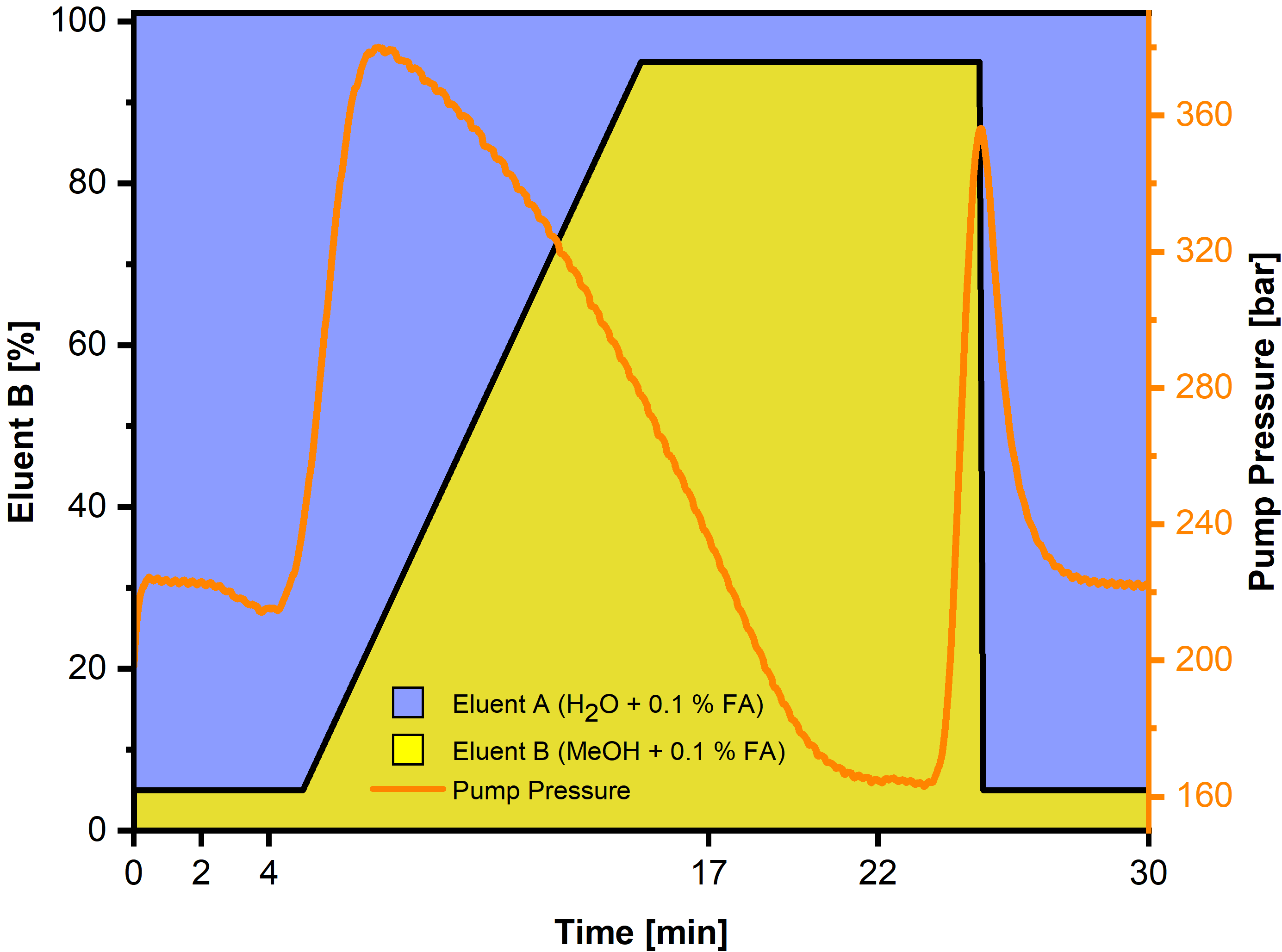


Figure SI-1: Water and methanol gradient, both spiked with 0.1 % formic acid (FA), over a run time of 30 min. In addition, information on the corresponding pump pressure is given. The flow rate was 0.3 mL/min.

Table SI-1: List of target chemicals including information on molecular formular and exact mass. Each compound was added in a concentration of 1 µg/L.

| **Compound** | **Molecular Formular** | **Exact Mass** |
| --- | --- | --- |
| Diclofenac | C_14_H_11_Cl_2_NO_2_ | 296.1528 |
| Carbamazepine | C_15_H_12_N_2_O | 236.2731 |
| 1H-Benzotriazole | C_6_H_5_N_3_ | 119.1258 |
| Diethyltoluamide | C_12_H_17_NO | 191.2731 |
| Metformin | C_4_H_11_N_5_ | 129.1648 |
| Gabapentin | C_9_H_17_NO_2_ | 171.2395 |
| Metoprolol | C_15_H_25_NO_3_ | 267.3684 |
| Acesulfame | C_4_H_4_KNO_4_S | 201.2441 |
| Hydrochlorothiazide | C_7_H_8_ClN_3_O_4_S_2_ | 297.7432 |
| Iopromide | C_18_H_24_I_3_N_3_O_8_ | 791.1039 |
| Valsartan | C_24_H_29_N_5_O_3_ | 435.5260 |

Table SI-2: List of isotopic labelled standards including information on molecular formular and exact mass. Each compound was added in a concentration of 1 µg/L.

| **Compound** | **Molecular Formular** | **Exact Mass** |
| --- | --- | --- |
| *Isotopic labelled standards* | | |
| Carbamazepine-D8 | C_15_D_8_H_4_N_2_O | 244.1445 |
| Sulfamethoxazole-D4 | C_10_D_4_H_7_N_3_O_3_S | 257.0765 |
| Metoprolol-D7 | C_30_D_14_H_36_N_2_O_6_ | 274.2272 |
| Diclofenac-^13^C_6_ | ^13^C_6_C_8_H_10_Cl_2_NNaO_2_ | 323.0180 |

Table SI-3: API-ESI source settings in positive and negative IM.

| **Atmospheric Pressure Electrospray Ionization Parameters** | | |
| --- | --- | --- |
| **Polarity** | positive | negative |
| **Transfer capillary temperature [°C]** | 320 | 360 |
| **Spray voltage [kV]** | 3.5 | 3.0 |
| **Sheath gas flow rate [a. u.]** | 37 | 40 |
| **Aux gas flow rate [a. u.]** | 15 | 15 |
| **Aux gas heater temperature [°C]** | 50 | 360 |
| **Sweep gas flow rate [a. u.]** | 1 | 0 |
| **S-lens RF level** | 50 | 50 |

Table SI-4: Information on full scan MS^1^ parameters.

| **Full‑MS‑Scan Parameters** | | |
| --- | --- | --- |
| **Modus** | positive | negative |
| **Resolution** | 70,000 | 70,000 |
| **AGC target** | 1,000,000 | 1,000,000 |
| **Maximum IT [ms]** | 100 | 50 |
| **Scan range** | 80 to 1000 m/z | 80 to 1000 m/z |

Table SI-5: msConvert software settings applied to convert raw chromatographic data from profile mode into .mzXML file format.

| **msConvert Options** | |
| --- | --- |
| Binary encoding precision | 64-bit |
| Write index | √ |
| Use zlib compression | √ |
| TPP compatibility | √ |
| **Centroidization** | |
| Filter | Peak Picking |
| Algorithm | Vendor |
| MS Levels | 1 - 2 |
| Min SNR | 0.1 |
| Min peak spacing | 0.1 |

## **MZmine3 parameters**

Table SI-6: MZmine3 settings for peak detection and alignment in positive ionization mode.

| Positive | **Parameters** | **Mass detection** | **Chromatogram building** | **Chromatogram deconvolution** | **Isotope pattern finder** | **Join aligner** | **Gap filling (peak finder)** | **Feature list blank subtraction** |
| --- | --- | --- | --- | --- | --- | --- | --- | --- |
| Peak Detection | Mass detector | Centroid |  |  |  |  |  |  |
|  | Noise level | 225000 |  |  |  |  |  |  |
|  | MS level | 1.0 |  |  |  |  |  |  |
|  | Min. group size in # of scans |  | 5 |  |  |  |  |  |
|  | Group intensity threshold |  | 450000 |  |  |  |  |  |
|  | Min height intensity |  | 1125000 |  |  |  |  |  |
|  | m/z tolerance |  | 0.005 Da or 5 ppm |  |  |  |  |  |
|  | Algorithm |  | | Local minimum search |  |  |  |  |
|  | Chromatographic threshold [%] |  |  | 85.0 |  |  |  |  |
|  | Minimum search range RT/Mobility [absolute] |  |  | 0.05 |  |  |  |  |
|  | Minimum relative height [%] |  |  | 0.0 |  |  |  |  |
|  | Minimum absolute height |  |  | 225000 |  |  |  |  |
|  | Min ratio of peak top/edge |  |  | 1.7 |  |  |  |  |
|  | Peak duration range [min] |  |  | 0.0-3.0 |  |  |  |  |
|  | Min # of data points |  |  | 5 |  |  |  |  |
|  | Chemical elements |  | | | H, C, N, O, S, Br |  |  |  |
|  | m/z tolerance |  |  |  | 0.001 Da or 5ppm |  |  |  |
|  | Maximum charge of isotope m/z |  |  |  | 1 |  |  |  |
|  | Search in scans |  |  |  | Single most intense |  |  |  |
| Peak Alignment and Filtering | m/z tolerance |  | | | | 0.0025 Da or 5 ppm | 0.0025 Da or 5 ppm |  |
|  | Weight for m/z tolerance |  |  |  |  | 70 |  |  |
|  | Retention time tolerance [absolute, min] |  |  |  |  | 0.3 | 0.3 |  |
|  | Weight for RT |  |  |  |  | 30 |  |  |
|  | Require same charge state |  |  |  |  | yes |  |  |
|  | Minimum data points |  |  |  |  |  | 1 |  |
|  | Intensity tolerance [%] |  |  |  |  |  | 30.00 |  |
|  | Minimum # of detection in blanks |  |  |  |  |  |  | 2 |
|  | Fold change increase |  |  |  |  |  |  | 300% |

Table SI-7: MZmine3 settings for peak detection and alignment in negative ionization mode.

| Negative | **Parameters** | **Mass detection** | **Chromatogram building** | **Chromatogram deconvolution** | **Isotope pattern finder** | **Join aligner** | **Gap filling (peak finder)** | **Feature list blank subtraction** |
| --- | --- | --- | --- | --- | --- | --- | --- | --- |
| Peak Detection | Mass detector | Centroid |  |  |  |  |  |  |
|  | Noise level | 50000 |  |  |  |  |  |  |
|  | MS level | 1.0 |  |  |  |  |  |  |
|  | Min. group size in # of scans |  | 5 |  |  |  |  |  |
|  | Group intensity threshold |  | 50000 |  |  |  |  |  |
|  | Min height intensity |  | 100000 |  |  |  |  |  |
|  | m/z tolerance |  | 0.005 Da or 5 ppm |  |  |  |  |  |
|  | Algorithm |  | | Local minimum search |  |  |  |  |
|  | Chromatographic threshold [%] |  |  | 85.0 |  |  |  |  |
|  | Minimum search range RT/Mobility [absolute] |  |  | 0.05 |  |  |  |  |
|  | Minimum relative height [%] |  |  | 0.0 |  |  |  |  |
|  | Minimum absolute height |  |  | 50000 |  |  |  |  |
|  | Min ratio of peak top/edge |  |  | 1.7 |  |  |  |  |
|  | Peak duration range [min] |  |  | 0.0-3.0 |  |  |  |  |
|  | Min # of data points |  |  | 5 |  |  |  |  |
|  | Chemical elements |  | | | H, C, N, O, S, Br |  |  |  |
|  | m/z tolerance |  |  |  | 0.001 Da or 5ppm |  |  |  |
|  | Maximum charge of isotope m/z |  |  |  | 1 |  |  |  |
|  | Search in scans |  |  |  | Single most intense |  |  |  |
| Peak Alignment and Filtering | m/z tolerance |  | | | | 0.0025 Da or 5 ppm | 0.0025 Da or 5 ppm |  |
|  | Weight for m/z tolerance |  |  |  |  | 70 |  |  |
|  | Retention time tolerance [absolute, min] |  |  |  |  | 0.3 | 0.3 |  |
|  | Weight for RT |  |  |  |  | 30 |  |  |
|  | Require same charge state |  |  |  |  | yes |  |  |
|  | Minimum data points |  |  |  |  |  | 1 |  |
|  | Intensity tolerance [%] |  |  |  |  |  | 30.00 |  |
|  | Minimum # of detection in blanks |  |  |  |  |  |  | 2 |
|  | Fold change increase |  |  |  |  |  |  | 300% |

## **Region of interest (ROI) approach**

Based on a set of three parameters, ROI filters[1] the signals from the datasets, avoiding background noise, and selects the signals that are more relevant: 1) a threshold intensity for MS signals that disregards instrumental noise; 2) a mass deviation based on the mass resolution used in the mass spectrometer for identifying different m/z values; 3) depending on the LC system and the MS detector speed, there is a minimum number of MS signals necessary to define a chromatographic peak. The detection of more compounds at lower concentrations or with poorer ionization efficiency may be possible with a lower threshold for signal intensity (e.g. 0.1% of maximum intensity). Conversely, setting intensity thresholds too low, such as 0.001% of maximum intensity, can lead to substantial experimental noise. This makes it challenging to differentiate between noise and analyte signals, especially when ionization is weak.

## **MCR-ALS Resolution of ROI LC-HRMS Data**

In the present study, MCR-ALS was used to resolve ROI LC-HRMS data from water samples in the mesocosm into their pure LC profiles and their mass spectra counterparts. The MCR-ALS data analysis process begins with an augmentation step. The global data matrix of several chromatographic runs along the suspected mode of breaking trilinearity is created. As a result, 53 samples were incorporated into a column-wise augmented matrix (**D**_aug_). Bilinear decomposition of each augmented data matrix Daug, containing K matrices, was performed simultaneously as follows[2,3]:

**D**_aug_= **C**_aug_ **S**^T^ + **E** (1)

Key parameters essential for optimizing solutions include the appropriate selection of components, initial estimate profiles, and the application of constraints. The initial estimation of component numbers for each LC window was based on a visual analysis of singular values from data matrices that surpassed noise levels through singular value decomposition (SVD). However, the chosen number of components was reevaluated based on the resulting MCR/ALS model performance depending on fitting error, percent of variance explained, model stability, and the reliability of the resolved chromatograms and MS spectra. The most popular approach for providing initial estimation of two-way data matrices, each of the **C** or **S**^T^, is employing SIMPLISMA[4] (simple interactive self-modeling mixture analysis). Then, bilinear decomposition of each **D**_aug_ is performed by iterative least squares minimization of ||**E**||, under constraints such as non-negativity of spectral and chromatographic profiles. To mitigate intensity ambiguities in the MCR model, the mass spectra of resolved components were normalized to spectra equal height constraint. The resolved mass spectra for common constituents in the various samples in **S**^T^ are enforced to be the same by column-wise augmentation, whereas the elution profiles in **C**_aug_ can differ (in retention time and peak shape) depending on the sample. Finally, by implementing the mentioned constraints and updating the **C**_aug_ and **S**^T^ profiles, the iterative optimization is continued, until the convergence criterion is fulfilled or limiting number of iterations is achieved. The quality of the results can be evaluated by the amounts of lack of fit (LOF) values and the percentage of explained variance (R^2^) as follows:

Lack of fit (%) = 100 × $\sqrt{\frac{\sum_{i,j} e_{ij}^{2}}{\sum_{i,j} d_{ij}^{2}}}$ (2)

R^2^=100 × $\frac{\sum_{i,j} d_{ij-}^{2}\sum_{i,j} e_{ij}^{2}}{\sum_{i,j} d_{ij}^{2}}$ (3)

where each d_ij_ shows each experimental data matrix and each e_ij_ is the residual element of **E** matrix. Following MCR-ALS processing of **D_aug_**, the area under the resolved chromatographic profile in **C**_aug_, were used to obtain the relative quantitative information of different compounds in the different types of water samples. To determine if the solutions provided by the decomposition are practical unique, the quality of the MCR-ALS modeling was also checked for rotational ambiguity. This was achieved through the implementation of the MCR-BAND software[5]. Finally, the calculated peak area under the resolved chromatographic profile in **C_aug_**, were used as the quantitative sores for follow-up multivariate data processing after a matrix cleaning and blank correction step [6].

1. **Multivariate Data Processing**

Principal Component Analysis (PCA) is widely used as an initial step in chemometric studies due to its ability to reveal general trends, specific data features, and potential outliers. As an unsupervised approach, PCA does not require predefined class labels, making it suitable for exploratory data analysis. The visualization primarily relies on scores and loading plots; however, for datasets with high dimensionality, interpreting these visualizations can become challenging.

**ASCA.** ASCA combines the variance-partitioning strength of Analysis of Variance (ANOVA) with the dimensionality-reduction capabilities of Simultaneous Component Analysis (SCA) [7]. A key consideration for ASCA is that the dataset must have a structured, designed format, where samples comprehensively represent all factor combinations (e.g., all time and treatment pairs). By using this analysis strategy, each experimental factor (and possible factor interactions) can be independently evaluated for its statistical significance. Mathematically, ASCA can be expressed as follows:

**X** = **1m**^T^ + **X**_F1_ + **X**_F2_ + **X** _(F1F2)_ + **E**  (4)

where 1m^T^ is the matrix of means, X_F1_ and X_F2_ are the effect matrices of the different factors F1 and F2 and X_(F1F2)_, describes the interaction between them. E is the residual matrix and contains the variation that cannot be described by the model. The effect matrix X_F1_ is decomposed as:

**X**_F1_ = **T**_F1_ **P**^T^_F1_ +**E**_F1_ (5)

In this expression, T_F1_ and P^T^_F1_​ represent the score and loading matrices for factor F1, while and E_F1_ ​ contains residuals. Scores and loadings enable visualization of patterns and relationships within the dataset, separating contributions from individual factors and their interactions. The significance of each factor is determined by evaluating the sum of squares of the effect matrices against their null hypothesis-based distribution, often estimated through randomization tests (6). In case of unbalanced designs, a modified version of ASCA, known as **ASCA+** is used[8]. As with many chemometric techniques, interpreting effect loadings becomes increasingly complex for datasets with a high number of variables. In this study, loadings were selected using bootstrapped confidence intervals. A comprehensive explanation of the ASCA method can be found in the literature (5).

**PLS-DA.** PLS-DA is a supervised multivariate method that integrates regression with discriminant analysis. It models the relationship between a matrix of predictor variables **X** (independent variables) and a response vector **y** (dependent variables) encoded as binary dummy variables (0 and 1) to represent class membership[9,10]. In a multiclass problem, dummy vector of **y** (N×1) is converted into a dummy matrix of **Y** (N×G), containing dummy codes 1 and 0 (G is the number of classes). In this method, PLS is first used to compute the scores matrix of **X**, i.e. **T**. Then, a linear discriminant analysis (LDA) model is built by using **T** as input. The resulting model can be visualize in the latent variable space that captures the most variance of **X** that is relevant to **y** or **Y**. In this study, PLS-DA was employed to identify variables of interest for each factor, such as location and time. Separate models were built for each factor, correlating sample peak areas with the corresponding class membership vector **y**. Model performance was validated using repeated Venetian blinds cross-validation, and the optimal number of latent variables (H) was determined by minimizing cross-validation classification errors and performing permutation tests. Additionally, PLS-DA facilitates feature ranking by calculating Variable Importance in Projection (VIP) scores. VIP scores for a given variable, j, across H latent variables a PLS1 model is calculated as follows:

VIP_j_=$\sqrt{p\frac{\sum_{h=1}^{H} w_{jk}^{2} . {Var}_{h}(y)}{\sum_{h=1}^{H} {Var}_{h}(y)}}$ (6)

Where *p* is the total number of predictor variables, $w$_jk_ is the weight of variable *j* for latent variable *h*. Var_h_​(y) is the variance in **y** explained by latent variable *h*. Hence, a variable with a higher VIP score has a greater impact on sample discrimination as a whole. These scores represent the weighted sum of squared PLS regression coefficients, reflecting each variable's contribution to the model. Commonly, variables with VIP scores exceeding 1 are considered significant, as their importance surpasses the average squared VIP value. However, this threshold can be adjusted depending on study objectives and data characteristics.

## **Initial parameter setting and correspondence**

Table SI-8: Various ROI & Mzmine3 feature extraction parameters in comparison applied to different quality control samples in positive ionization mode. In addition to the absolute number of extracted ROIs & features, information on the inclusion of targets is provided.

| **Sample** |  | **ROI#30** | **MZmine3 standard after ADAP** | **MZmine3 standard after Deconvolution** | **MZmine3 harmonised 30*5 after ADAP** | **MZmine3 harmonised 30*5 after Deconvolution** |
| --- | --- | --- | --- | --- | --- | --- |
| QC samples | Targets | **7 out of 7** | 7 out of 7 | 7 out of 7 | 0 out of 7 | 0 out of 7 |
|  | # ROIs  /Features | **371** | 239 | 336 | 107 | 158 |
|  | Overlap |  | 163/208/31 | 307 | 264/107/0 | 158 |
|  |  |  | 40.5%/51.7%/7.7% |  | 71%/29%/0% |  |
|  |  |  | 87% of features are covered | 91% of features are covered | 100% of features are covered | 100% of features are covered |
|  |  |  |  |  |  |  |
| **Sample** |  | **ROI#15** | **MZmine3 standard after ADAP** | **MZmine3 standard after Deconvolution** | **MZmine3 harmonised 15*5 after ADAP** | **MZmine3 harmonised 15*5 after Deconvolution** |
| QC samples | Targets | **7 out of 7** | 7 out of 7 | 7 out of 7 | 0 out of 7 | 0 out of 7 |
|  | # ROIs  /Features | **570** | 239 | 336 | 240 | 306 |
|  | Overlap |  | 339/231/8 | 328 | 334/236/4 | 303 |
|  |  |  | 58.7%/40%/1.4% |  | 58%/42%/0% |  |
|  |  |  | 97% of features are covered | 98% of features are covered | 98% of features are covered | 99% of features are covered |

Table SI-9: Various ROI & Mzmine3 feature extraction parameters in comparison applied to different quality control samples in negative ionization mode. In addition to the absolute number of extracted ROIs & features, information on the inclusion of targets is provided.

| **Sample** |  | **ROI#30** | **MZmine3 standard after ADAP** | **MZmine3 standard after Deconvolution** | **MZmine3 harmonised 30*5 after ADAP** | **MZmine3 harmonised 30*5 after Deconvolution** |
| --- | --- | --- | --- | --- | --- | --- |
| QC samples | Targets | 4 out of 4 | 4 out of 4 | 4 out of 4 | 0 out of 4 | 0 out of 4 |
|  | # ROIs  /Features | 1188 | 1389 | 1385 | 533 | 702 |
|  | Overlap |  | 222/966/423 | 1114 | 676/512/21 | 671 |
|  |  |  | 14%/60%/26% |  | 56%/42%/2% |  |
|  |  |  | 70% of features are covered | 80% of features are covered | 96% of features are covered | 96% of features are covered |
|  |  |  |  |  |  |  |
| **Sample** |  | **ROI#15** | **MZmine3 standard after ADAP** | **MZmine3 standard after Deconvolution** | **MZmine3 harmonised 15*5 after ADAP** | **MZmine3 harmonised 15*5 after Deconvolution** |
| QC samples | Targets | 4 out of 4 | 4 out of 4 | 4 out of 4 | 0 out of 4 | 0 out of 4 |
|  | # ROIs  /Features | 1606 | 1389 | 1385 | 812 | 988 |
|  | Overlap |  | 456/1150/239 | 1222 | 833/773/39 | 934 |
|  |  |  | 25%/62%/13% |  | 51%/47%/2% |  |
|  |  |  | 83% of features are covered | 88% of features are covered | 95% of features are covered | 95% of features are covered |

At this stage of data processing, an important consideration arises within MZmine3 that must be accounted for in all subsequent comparisons in this study. Specifically, during peak deconvolution, the extracted XICs are examined for the presence of one or more peaks within each XIC. The resulting features exhibit nearly identical masses, and the initial one-to-one correspondence between ROIs and XICs is no longer maintained. For the QC sample discussed above: While the proportion of overlapping ROIs remains unchanged, the detection of multiple peaks within individual overlapping XICs potentially leads to an increase in the relative correspondence of features from 87% to 91% in the positive IM, and from 70% to 80% in the negative IM.

## **MCR/ALS resolution: Example**
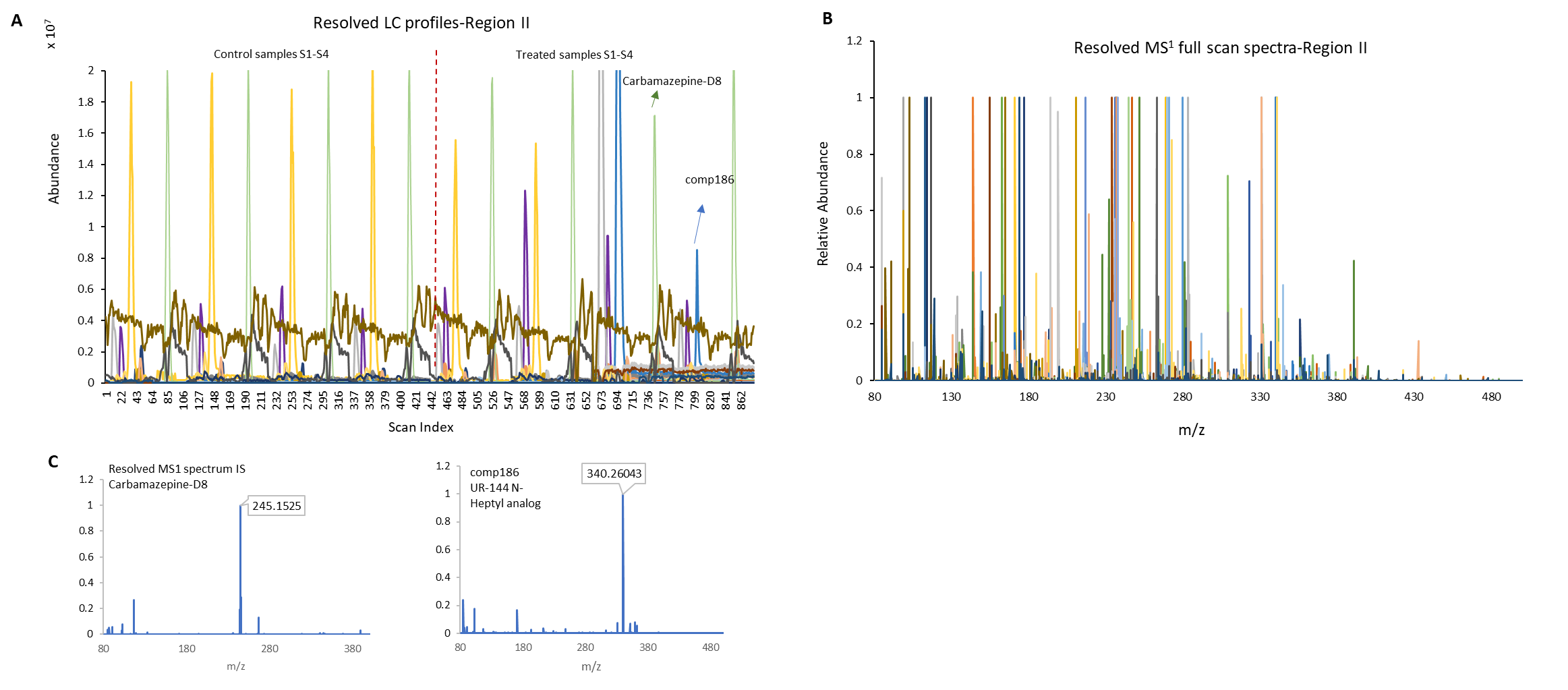


Figure SI-2: Representation of MCR/ALS resolved chromatographic (A) and mass spectral (B) profiles of 35 components retrieved in the second chromatographic region for one sub-set of replicate control and treated samples from S1 to S4, which ranged from 9 to 11.45 min. Resolved LC and MS1 profiles of carbamazepine-D8 (internal standard) and a sample type-dependent component (Nr. 186) is presented in panels A, B, C and D. This component with [M+H]+=340.2604 was tentatively identified as UR-144 N-Heptyl analog.

## **Overview on data reduction and organization throughout this study**


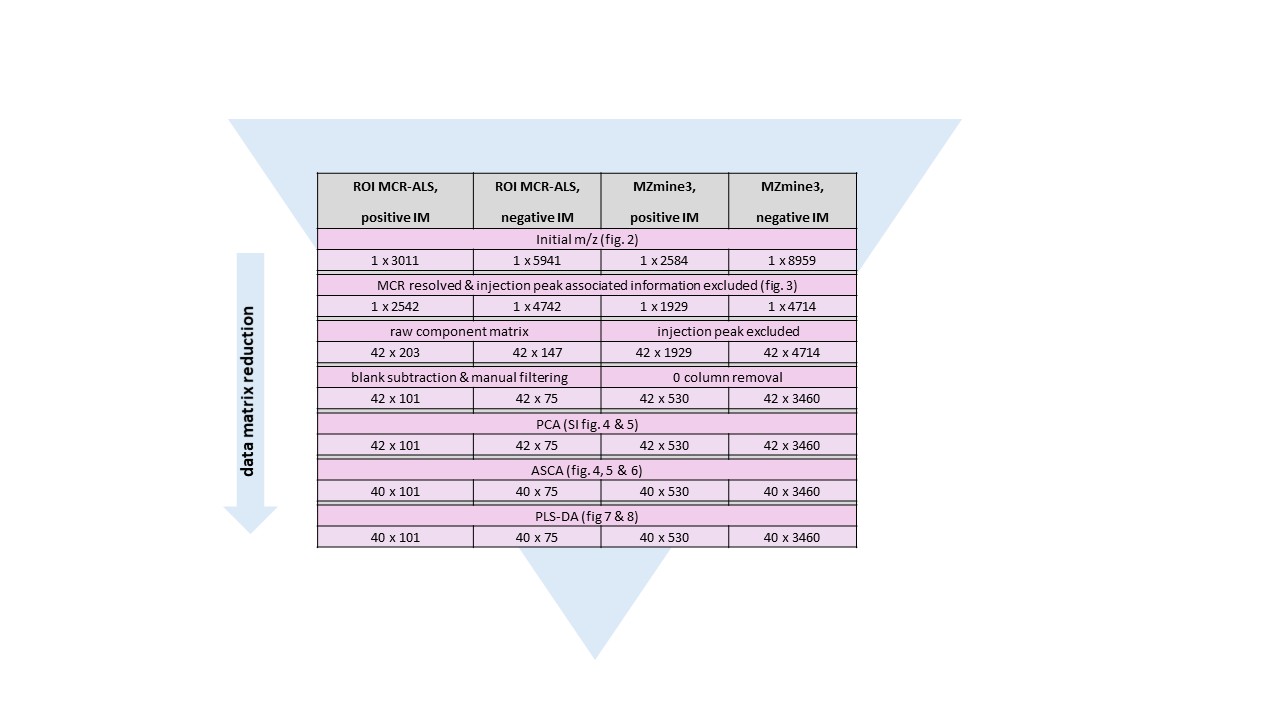


Figure SI-3: Overview on the data structure reduction throughout the process. Detailed information on how many features and components are considered in different chemometric approaches are given.

## **Supporting results for PCA, ASCA and PLS-DA**
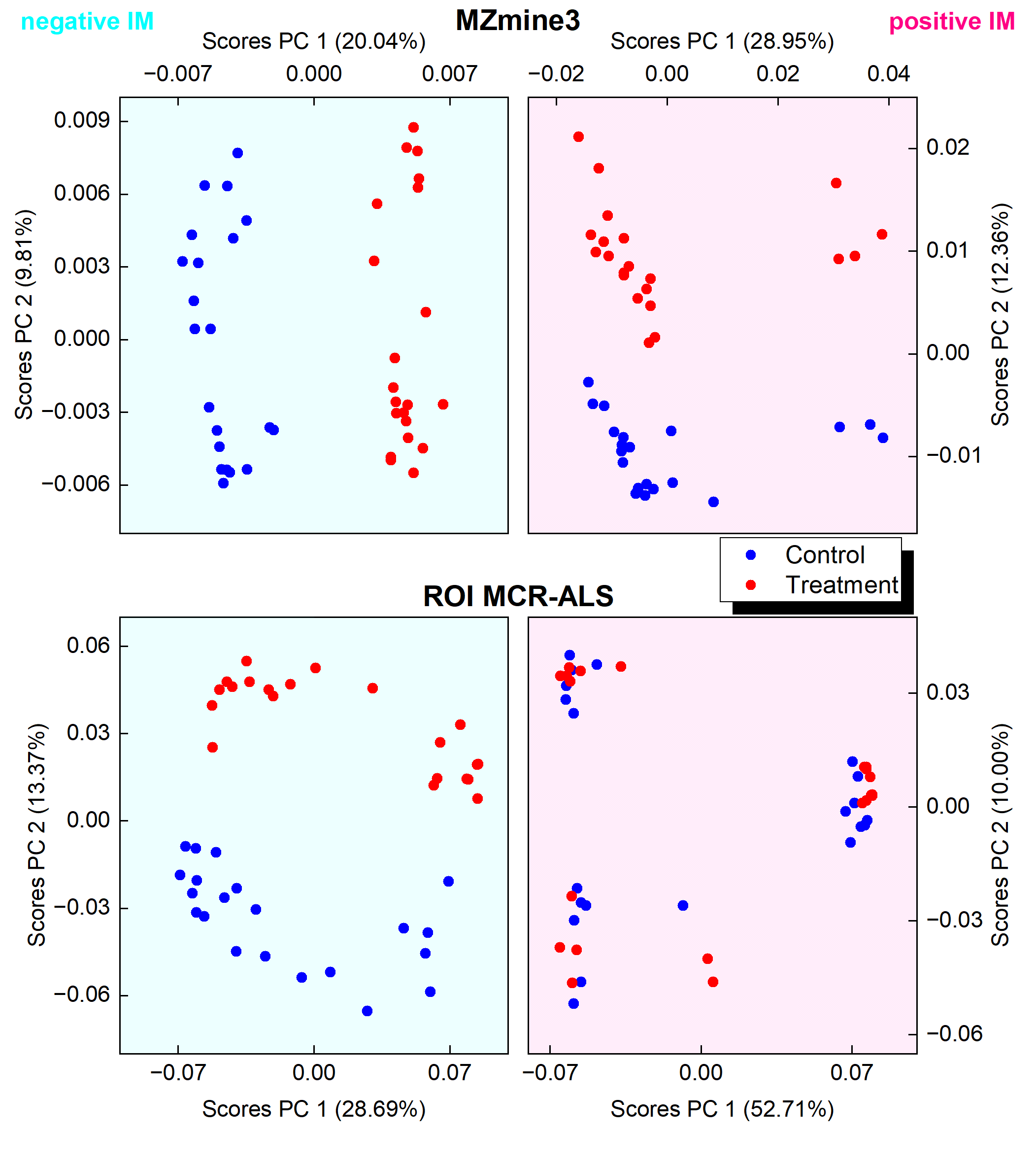


Figure SI-4: Scores on PC 1 & 2 obtained from PCA of feature and component list by MZmine3 and MCR/ALS in both IMs. The color code indicates sample types, control (blue) and treatment (red).


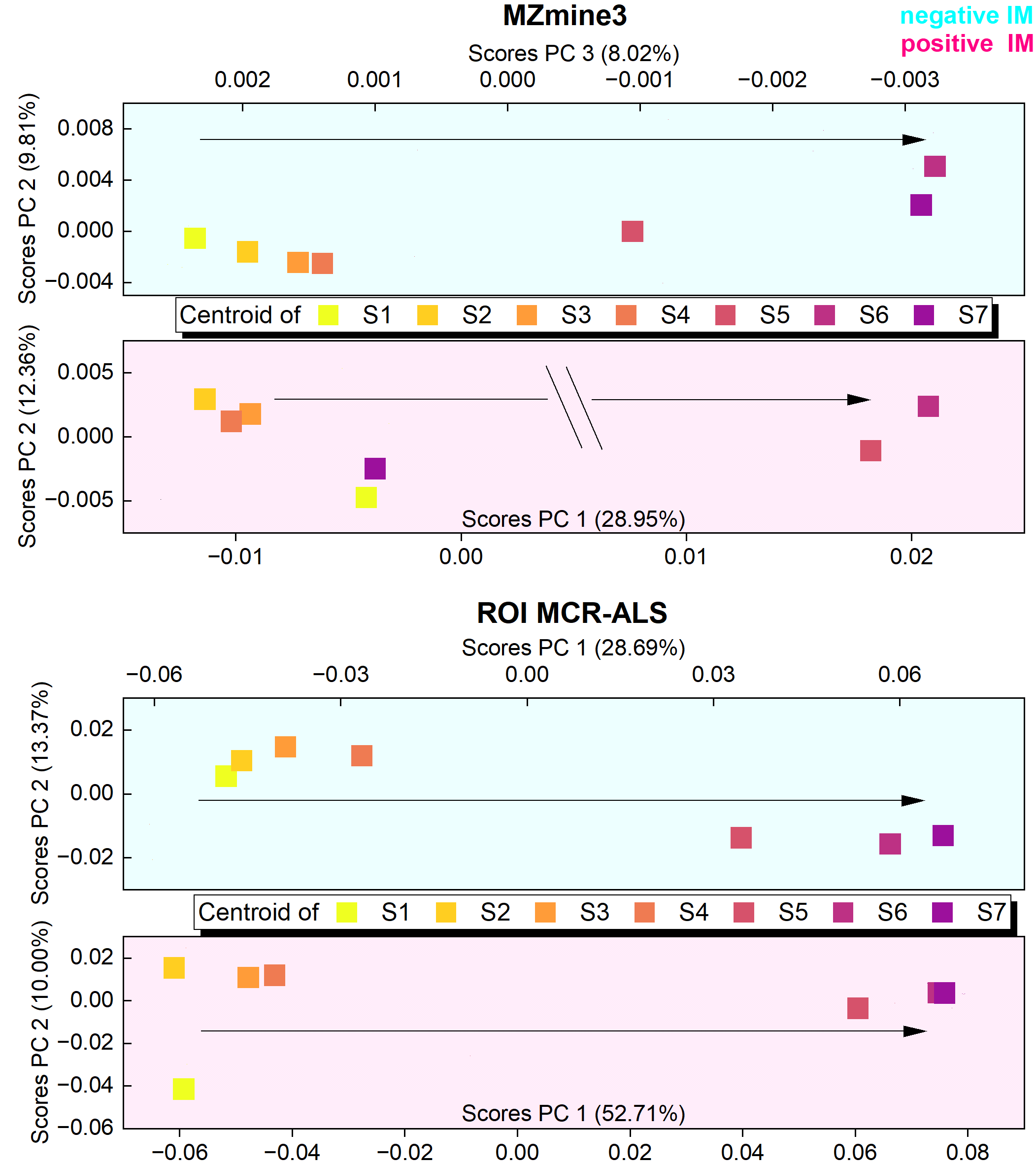


Figure SI-5: Representation of temporal variability in PCA subspaces for MZmine3 and MCR/ALS data in both IMs. The samples taken at the same time are visually connected to their mean. The color code indicates time series of sampling (S1 - S7).

In addition to different sample types, the experimental design involves a temporal dimension. Principal components were then selected to elaborate the time series in order to verify whether a continuous time trend exists. The centroids of various samples were visualized for this purpose. For ROI MCR/ALS, in spite of MZmine3 data, the arrangement of centroids along the first PC already unravels the time series of sampling in both IMs. Here, it was hypothesized that the temporal effect may be a major factor in diverting chemical profiles because it contributes significantly to the majority of PC1 variations for majority of data matrices.


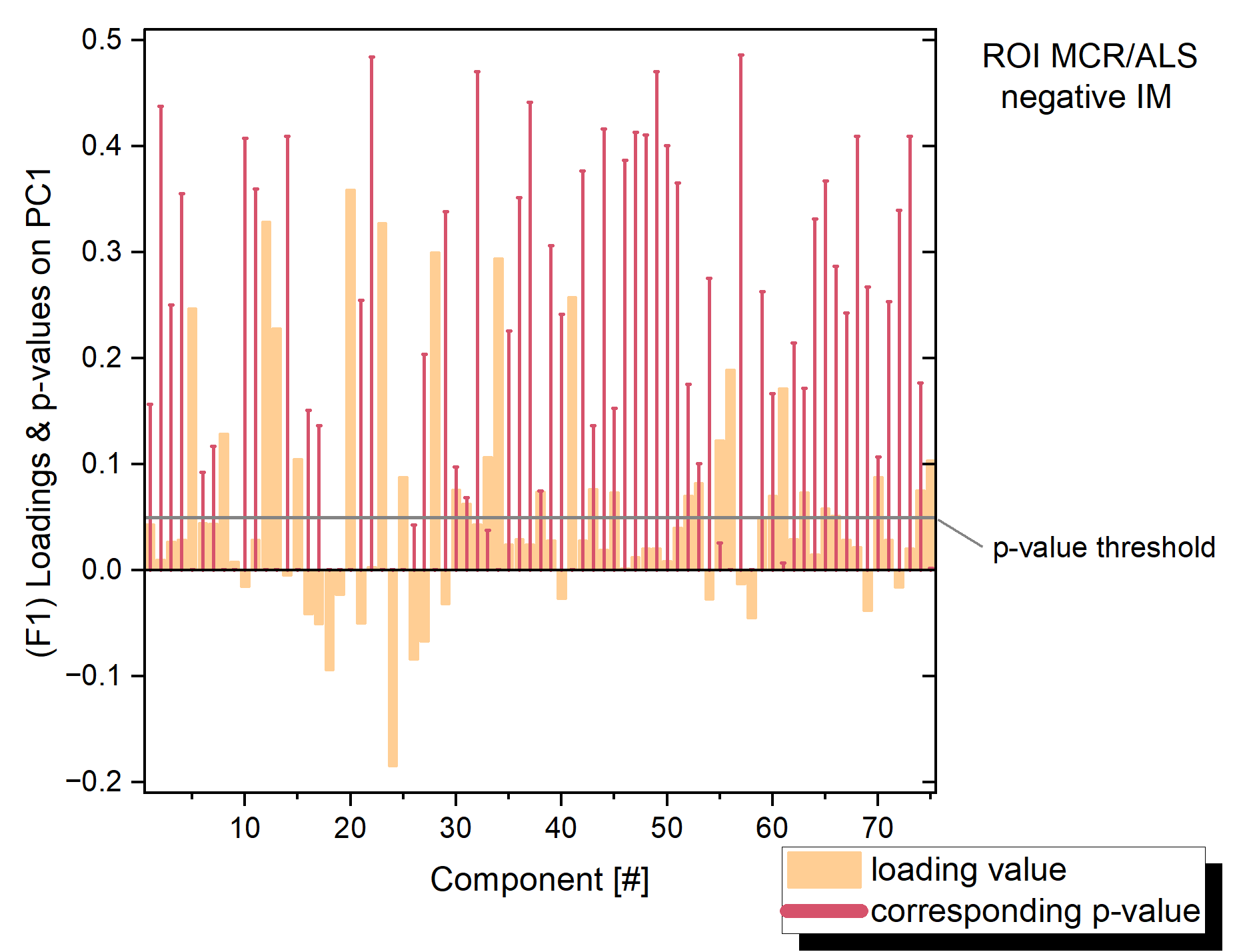


Figure SI-6: An exemplary representation of how the respective components (for ROIMCR negative data) were filtered based on their loading values and associated p-values. The loading values are depicted as sand-colored columns, while the p-values are illustrated as reddish-brown bars.

Table SI-10: Tabular listing of the absolute number of ROIs and features prioritized based on bootstrapped loading values, depending on the ionization mode. Additionally, the number of corresponding ROIs or features is provided under the "overlap" column.

| *Ionization* | *positive* | | | *negative* | | |
| --- | --- | --- | --- | --- | --- | --- |
| **DoE** | **#ROI** | **#overlap** | **#Feature** | **#ROI** | **#overlap** | **#Feature** |
| **Factor 1** | 75 | 11 | 66 | 619 | 298 | 1397 |
| **Factor 2** | 249 | 24 | 245 | 736 | 371 | 1948 |


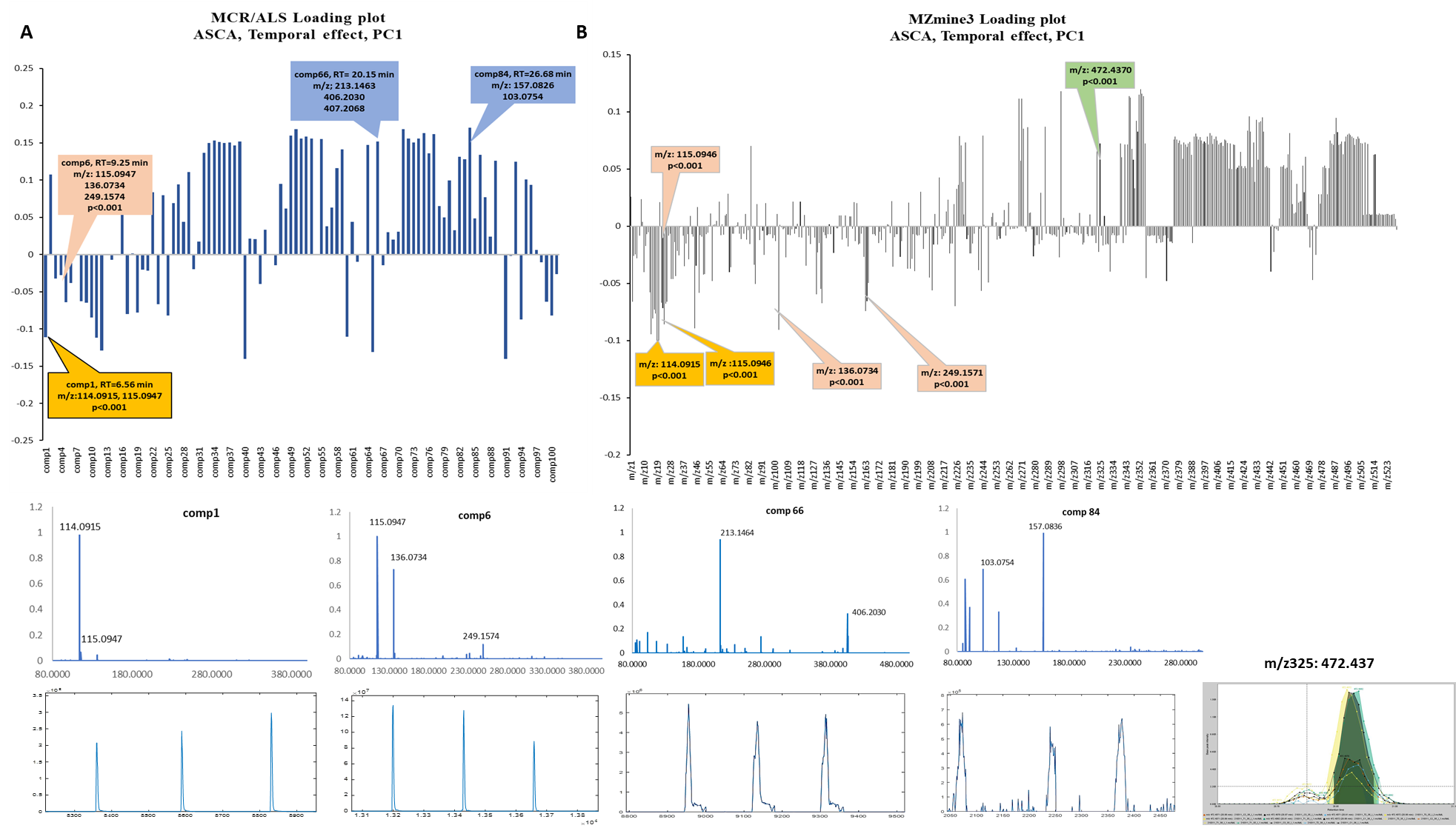


Figure SI-7: Representation of loading plots for first PC of ASCA models, temporal effects, for (A) ROIMCR and (B) MZmine3 datasets. M/z features related to comps 1 and 6 are shared between two workflows. Comps 66 and 84 are ROIMCR’s unique features and m/z325 is Mmine3’s unique feature.

Table SI-11:: PLS-DA model performance parameters broken down by design of experiment factors (sample type & time).

| 2-class PLS-DA models | | | | | | |
| --- | --- | --- | --- | --- | --- | --- |
| *Sample type classification* | ***Latent variables*** | ***Class error rate (CV)*** | ***(X) Explained variance (%)*** | ***R^2^ (CV)*** | ***Probability of model insignificance*** |  |
| *ROI MCR/ALS positive* | 2 | 0.07 | 47.1 | 0.605 | <0.01 |  |
| *ROI MCR/ALS negative* | 2 | 0.000 | 41.8 | 0.940 | <0.01 |  |
| *MZmine3 positive* | 2 | 0.000 | 40.6 | 0.926 | <0.01 |  |
| *MZmine3 negative* | 2 | 0.000 | 39.3 | 0.951 | <0.01 |  |
| *Time classification* |  | | | | |  |
| *ROI MCR/ALS positive* | 2 | 0.028 | 61.3 | 0.945 | <0.01 |  |
| *ROI MCR/ALS negative* | 2 | 0.028 | 46.8 | 0.829 | <0.01 |  |
| *MZmine3 positive* | 3 | 0.028 | 56.1 | 0.770 | <0.01 |  |
| *MZmine3 negative* | 3 | 0.028 | 56.7 | 0.819 | <0.01 |  |
| 3-class PLS-DA models | | | | | | |
| *Sample type & time classification* | ***Latent variables*** | ***Class error rate (CV)*** | ***(X) Explained variance (%)*** | ***R^2^ (CV)*** | ***Probability of model insignificance*** |  |
| *ROI MCR/ALS positive* | 4 | 0.054  0.000  0.028 | 73.6 | 0.715  0.820  0.941 | <0.01 |  |
| ROI MCR/ALS negative | 2 | 0.142  0.042  0.056 | 53.7 | 0.398  0.683  0.778 | <0.01 |  |
| MZmine3 positive | 3 | 0.160  0.183  0.106 | 59.0 | 0.328  0.360  0.753 | <0.01 |  |
| MZmine3 negative | 3 | 0.017  0.042  0.056 | 53.5 | 0.407  0.516  0.776 | <0.01 |  |
| - $\mathbf{Class Err. =}$average of false positive rate and false negative rate for class   = 1 – $\frac{\mathbf{(sensitivity + specificity)}}{\mathbf{2}}$   - Cross validation: venetian blinds - Probability of model insignificance vs. permuted samples: random t-test. Values less than 0.05 indicate the model is significant at the 95 % confidence level. | | | | | | |

**References**

1. Gorrochategui E, Jaumot J, Tauler R (2019) ROIMCR: a powerful analysis strategy for LC-MS metabolomic datasets. BMC Bioinformatics 20 (1):256. doi:10.1186/s12859-019-2848-8

2. Tauler R (1995) Multivariate curve resolution applied to second order data. Chemometrics and Intelligent Laboratory Systems 30 (1):133--146. doi:10.1016/0169-7439(95)00047-X

3. Tauler R, Maeder M, de Juan A (2009) Multiset Data Analysis: Extended Multivariate Curve Resolution. In: Brown SD, Tauler R, Walczak BBTCC (eds) Comprehensive Chemometrics. Elsevier, Oxford, pp 473--505. doi:10.1016/B978-044452701-1.00055-7

4. Windig W, Guilment J (1991) Interactive self-modeling mixture analysis. Analytical Chemistry 63 (14):1425--1432

5. Jaumot J, Tauler R (2010) MCR-BANDS: A user friendly MATLAB program for the evaluation of rotation ambiguities in Multivariate Curve Resolution. Chemometrics and Intelligent Laboratory Systems 103 (2):96--107

6. Hohrenk LL, Vosough M, Schmidt TC (2019) Implementation of Chemometric Tools To Improve Data Mining and Prioritization in LC-HRMS for Nontarget Screening of Organic Micropollutants in Complex Water Matrixes. Analytical Chemistry 91 (14):9213-9220. doi:10.1021/acs.analchem.9b01984

7. Smilde AK, Jansen JJ, Hoefsloot HC, Lamers RJ, van der Greef J, Timmerman ME (2005) ANOVA-simultaneous component analysis (ASCA): a new tool for analyzing designed metabolomics data. Bioinformatics 21 (13):3043-3048. doi:10.1093/bioinformatics/bti476

8. Thiel M, Féraud B, Govaerts B (2017) ASCA+ and APCA+: Extensions of ASCA and APCA in the analysis of unbalanced multifactorial designs. Journal of Chemometrics 31 (6). doi:10.1002/cem.2895

9. Lee LC, Liong CY, Jemain AA (2018) Partial least squares-discriminant analysis (PLS-DA) for classification of high-dimensional (HD) data: A review of contemporary practice strategies and knowledge gaps. vol 143. Royal Society of Chemistry. doi:10.1039/c8an00599k

10. Barker M, Rayens W (2003) Partial least squares for discrimination. Journal of Chemometrics 17 (3):166--173. doi:10.1002/cem.785
